# Glucose Starvation-Induced Dispersal of *Pseudomonas aeruginosa* Biofilms Is cAMP and Energy Dependent

**DOI:** 10.1371/journal.pone.0042874

**Published:** 2012-08-14

**Authors:** Tran T. Huynh, Diane McDougald, Janosch Klebensberger, Budoor Al Qarni, Nicolas Barraud, Scott A. Rice, Staffan Kjelleberg, David Schleheck

**Affiliations:** 1 Centre for Marine Bio-Innovation, School of Biotechnology and Biomolecular Science, University of New South Wales, Sydney, Australia; 2 Advanced Environmental Biotechnology Centre, Nanyang Technological University, Singapore, Singapore; 3 The Singapore Centre on Environmental Life Sciences Engineering, Nanyang Technological University, Singapore, Singapore; 4 Department of Biological Sciences and Research School Chemical Biology, University of Konstanz, Konstanz, Germany; National Institutes of Health, United States of America

## Abstract

Carbon starvation has been shown to induce a massive dispersal event in biofilms of the opportunistic pathogen *Pseudomonas aeruginosa*; however, the molecular pathways controlling this dispersal response remain unknown. We quantified changes in the proteome of *P. aeruginosa* PAO1 biofilm and planktonic cells during glucose starvation by differential peptide-fingerprint mass-spectrometry (iTRAQ). In addition, we monitored dispersal photometrically, as a decrease in turbidity/opacity of biofilms pre-grown and starved in continuous flow-cells, in order to evaluate treatments (e.g. inhibitors CCCP, arsenate, chloramphenicol, L-serine hydroxamate) and key mutants altered in biofilm development and dispersal (e.g. *nirS*, *vfr*, *bdlA*, *rpoS, lasRrhlR*, Pf4-bacteriophage and *cyaA*). In wild-type biofilms, dispersal started within five minutes of glucose starvation, was maximal after 2 h, and up to 60% of the original biomass had dispersed after 24 h of starvation. The changes in protein synthesis were generally not more than two fold and indicated that more than 100 proteins belonging to various classes, including carbon and energy metabolism, stress adaptation, and motility, were differentially expressed. For the different treatments, only the proton-ionophore CCCP or arsenate, an inhibitor of ATP synthesis, prevented dispersal of the biofilms. For the different mutants tested, only *cyaA*, the synthase of the intracellular second messenger cAMP, failed to disperse; complementation of the *cyaA* mutation restored the wild-type phenotype. Hence, the pathway for carbon starvation-induced biofilm dispersal in *P. aeruginosa* PAO1 involves ATP production via direct ATP synthesis and proton-motive force dependent step(s) and is mediated through cAMP, which is likely to control the activity of proteins involved in remodeling biofilm cells in preparation for planktonic survival.

## Introduction

Surface-adhered communities of microorganisms are embedded in a matrix composed of extracellular polymeric substances (EPS) and water [1], [2]. Such biofilm communities contribute to serious health problems in humans, and are difficult to eradicate as they exhibit substantially increased resistance to antimicrobials [3]. Bacterial biofilm formation proceeds through several distinct stages, with dispersal being the final stage of the biofilm life cycle [4]–[6]. The dispersal of cells from a biofilm is crucial for the colonization of new niches and broadly for species in the community to survive. Therefore, understanding the dispersal stage of the biofilm life cycle has relevance for prevention, control and removal of biofilms in both industrial and medical settings. Several mechanisms contribute to dispersal, including decreases in bacterial adhesiveness and degradation of the biofilm matrix [7], [8], environmental cues, e.g. changes in levels of oxygen [9], iron [10], and nutrients [11]–[13]. Starvation (carbon, nitrogen, or oxygen) can also induce biofilm dispersal in multiple species [6], [14]–[16]; however, the molecular pathways that trigger dispersal remain unclear. In the case of glucose starvation-induced dispersal in *Pseudomonas aeruginosa*, starvation leads to a reduction of intracellular levels of c-di-GMP which is linked to dispersal [15]. For biofilms of *Pseudomonas putida*, the glucose depletion induced a decrease in c-di-GMP levels, via an unknown pathway, leading to dissociation of c-di-GMP from a transmembrane protein LapD, which controls the activity of the periplasmic protease LapG. Under low levels of c-di-GMP, repression of LapG by LapD is alleviated, resulting in cleavage of the anchor protein LapA and dissociation of the biofilm [17].

The intracellular signaling molecule, nitric oxide (NO) disperses *P. aeruginosa* PAO1 biofilms at low, non-toxic concentrations (nanomolar) [18]–[22]. The NO signaling pathway regulates c-di-GMP levels where sensing of NO leads to a decrease in intracellular c-di-GMP levels [21] and NO-mediated dispersal is dependent on the chemotaxis regulator BdlA [18].

While there is a common theme that links dispersal with c-di-GMP [23], dispersal is clearly a complex process involving a range of cues, signals, intracellular second messengers and effectors, and the pathways that link the many different dispersal-inducing cues with effectors may be equally complex. Dispersal has also been linked to bacteriophage activity, global regulators of stress/starvation adaptation, cell-cell signaling systems, enzymatic activity and surface active molecules [23]–[25].

In this study, *P. aeruginosa* PAO1 was used as model organism for a further assessment of its biofilm-dispersal response after glucose starvation and investigation of the underlying mechanisms. Proteomic analysis indicated broad changes in protein synthesis, which argues for the role of global regulators of gene expression and protein activity. We found that cAMP biosynthesis via *cyaA* was required for biofilm dispersal under glucose starvation. Further, by treating the biofilms with a proton-ionophore CCCP, or a phosphate analog arsenate, and consequently inhibiting proton-motive force and energy production, we demonstrated that the dispersal of cells from biofilms was abolished. The role of cAMP was supported by treatment with atropine which altered dispersal and by measurements of cAMP in CCCP, arsenate and atropine treated cells and the *cyaA* mutant.

## Results

### Quantification of glucose starvation-induced biofilm dispersal

**Figure 1:**
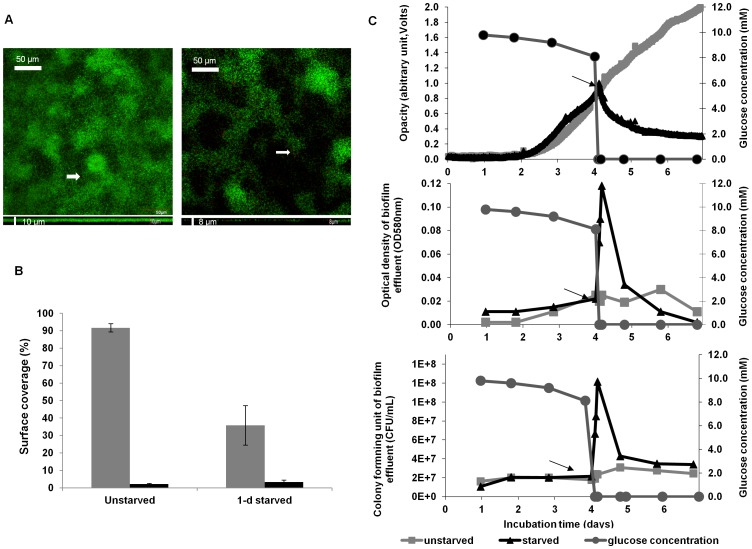
Glucose starvation-induced biofilm dispersal of *P. aeruginosa*. Dispersal of *P. aeruginosa* PAO1 WT biofilms grown in continuous-flow cells was assessed by confocal microscopy and image analysis (A, B), and by biofilm-opacity monitoring using a photometrical device (C). The biofilms were pre-grown under a continuous flow of glucose/M9-salts medium (100 µM CaCl_2_) and glucose starvation was induced at the time indicated (C, arrow). (A) Representative CLSM images of *P. aeruginosa* PAO1 MA67 WT biofilms stained with the LIVE/DEAD *BacLight* bacterial viability kit (Molecular Probes Inc., Eugene, OR, USA) after 4 days (left) and after 1 day glucose starvation (right) are shown in CSLM X-Y images (top) and X-Z images (bottom). (B) Percent surface coverage as determined by ImageJ analysis of live (grey) and dead (black) cells of a 4 day-old biofilm of *P. aeruginosa* before and 24 h after glucose starvation. The error bars represent standard errors (n = 3). (C) Representative data obtained by continuous photometrical biofilm-density measurement (top graph) of a biofilm during growth and starvation (black) in comparison to an unstarved control biofilm (grey). The glucose concentration in the effluent was determined as an indicator of the starvation event (circles). Dispersed cells in the effluent were determined as optical density (OD_580 nm_) (center graph) and CFU (bottom graph).

The dispersal of *P. aeruginosa* biofilms during glucose starvation was quantified, firstly, when biofilms were pre-grown in standard continuous-flow cells for confocal laser scanning microscopic (CLSM) analysis. Starvation was applied by switching the medium flow from glucose/M9-salts medium to glucose-free M9-salts medium, and the biofilms were stained and imaged using CLSM in comparison to unstarved control biofilms. Secondly, the flow-cell setup was modified to include a photometrical device that quantified the biofilm biomass continuously during the starvation event, by using the ‘biofilm opacity’ (determined as OD_580 nm_) as a proxy for total biomass (see Material and Methods and [15]). In the example shown (Fig. 1), glucose starvation was applied to a 4-day old biofilm. Analysis of CLSM images indicated that on average 60% of the original biofilm biomass had dispersed after 24 h of starvation (Fig. 1AB). Furthermore, the CLSM image analysis of the live-and-dead stained biofilm indicated that the number of dead cells was not increased in the biofilm remaining after the glucose starvation and dispersal event (Fig. 1A). This suggested that starvation-induced dispersal was not linked to loss of viability. The use of the continuous-flow setup with biofilm-opacity measurement revealed that biofilms of *P. aeruginosa* PAO1 WT dispersed within a few minutes of being exposed to glucose starvation, as indicated by a rapid decrease in the biofilm opacity readings (Fig. 1C). Results of three independent experiments showed a consistent, rapid dispersal of starved biofilms where biofilm density was reduced on average 44% (varied from 28–59%) after 1 day of starvation (data not shown). The dispersal event was confirmed by determination of the OD_580 nm_ and CFUs of the effluent; the glucose concentration was also monitored before and after glucose starvation to confirm the time point of glucose depletion (Fig. 1C). At the time glucose levels dropped, the OD of the effluent increased 3-fold within 5 min of starvation, and reached a maximum value after 2 h (5-fold, from OD_580 nm_ 0.02 to 0.12) (Fig. 1C), indicating that rapid dispersal occurred. Correspondingly, CFUs increased from approximately 2×10^7^ CFU ml^−1^ before starvation to 1×10^8^ CFU ml^−1^ after 40 min of starvation, and by 24 h had returned to pre-starvation levels. Microscopic inspection of the effluent (5, 30, 60, 90 min, 4 h and 1 day after starvation) revealed that the dispersing cells were single cells rather than clumps of cells, indicating that sloughing was not occurring in our growth system (data not shown). Finally, control biofilms not subjected to starvation exhibited a linear increase in biofilm opacity, reflecting continued growth of the biofilm, and steadily low OD and CFUs of the effluent, reflecting a basal, ‘seeding dispersal’ of growing biofilm (Fig. 1C).

### Characterization of the dispersal proteome

**Figure 2:**
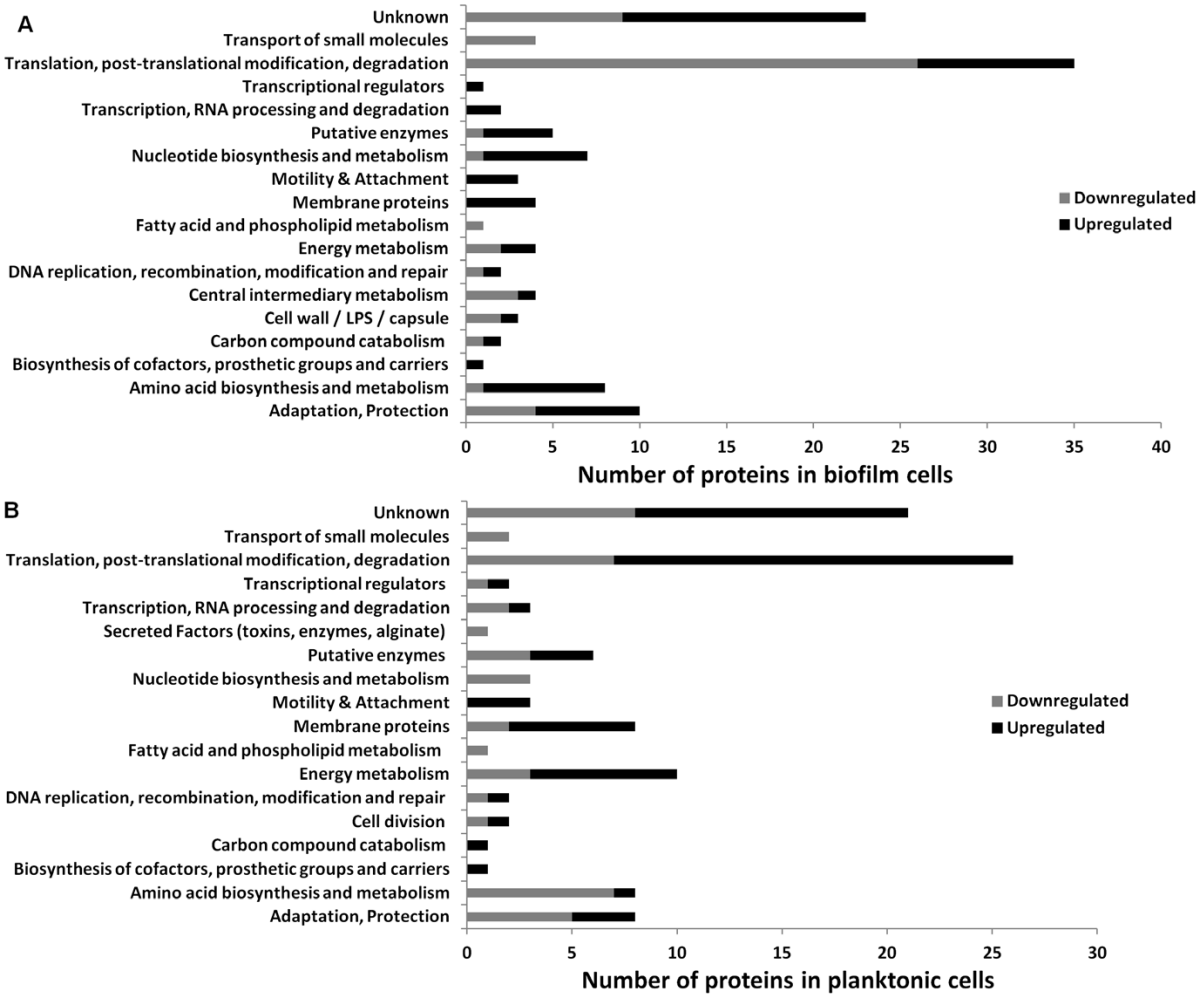
Functional annotation of differentially expressed proteins. Proteins that were differentially expressed in *P. aeruginosa* starved biofilms were compared to unstarved biofilm (A) and dispersal cells (B). The functional categories are according to *Pseudomonas* Community Annotation Project (www.pseudomonas.com).

iTRAQ was used to quantitatively compare protein synthesis of *P. aeruginosa* biofilm and dispersal cells in response to glucose starvation. Therefore, biofilms were pre-grown and starved in the flow cells, and the starvation event monitored, as described above. The biofilm biomass was harvested from the silicon tubing attached to the biofilm flow cells (i.e. from waste medium line; see Material and Methods) in order to provide sufficient biomass for analysis. Furthermore, the effluent samples were collected for 2 h before starvation and for 2 h after initiation of starvation. The experiment was performed twice with similar results. Figure 2 summarizes the classes of proteins that were differentially expressed in the biofilm cells and in the planktonic cells which were collected from the dispersing population. The complete list of differentially expressed proteins is presented in supplementary data as proteins in biofilms (Table S1) and in planktonic cells from the effluent (Table S2) before and after starvation, and proteins that were differentially expressed in both biofilm and planktonic cells (Table S3).

Protein synthesis was compared between starved and unstarved conditions for both the biofilm and planktonic populations. For both populations, 450 proteins were differentially expressed upon induction of starvation and of those, 118 and 109 were significantly different (*p*<0.05) from the unstarved population of biofilms (Table S1) and planktonic cells (Table S2) respectively. These proteins belonged to 17 functional groups (Fig. 2) including translation, post-translational modification and degradation, metabolic processes, such as amino acid metabolism, carbon catabolism, cofactor biosynthesis, motility and attachment, membrane proteins, adaptation and protection as well as unknown proteins and putative enzymes. Twenty-three proteins were differentially expressed in both starved biofilm and planktonic populations. Among them, only two proteins (succinate dehydrogenase and peptidoglycan-associated lipoprotein) were upregulated in both populations. The remaining 21 proteins were inversely up or down-regulated in the two populations (Table S3). This response highlights the different physiologies of sessile vs. planktonic cells.

In planktonic cells, 10 proteins involved in energy metabolism were detected; three were down-regulated (fumarate hydratase class II 1, probable malate:quinine, succinyl-CoA ligase [ADP-forming] subunit alpha) and seven were up-regulated (ATP synthase delta chain, azurin, nitrite reductase, polyhydroxyalkanoate synthesis protein PhaF, probable cytochrome c, probable outer membrane protein, succinate dehydrogenase). Components of chemotactic signal transduction systems, the fimbrial assembly proteins PilQ and PilM, were up-regulated in planktonic cells. From the starved biofilm cells, fimbrial protein PilA which is a component of type IV pili [26], motility protein FimV and B-type flagellin FliC were all upregulated (1.3-fold); whereas fimbrial protein PilQ and PilM were found to be upregulated in the starved planktonic cells (1.9-fold and 1.7-fold, respectively). Attachment proteins differentially expressed in planktonic cells include the upregulated outer membrane proteins OprG, porin F and peptidoglycan-associated lipoprotein; and down-regulated proteins including insulin-cleaving metalloproteinase. For biofilm samples, pyoverdine biosynthesis protein PvdE which is also an iron-related protein was upregulated 4-fold. Proteins involved in adaptation and protection that were differentially regulated in biofilm cells include bacterioferritin (down-regulated 1.33-fold). Proteins, such as chemotactic transducer PctB, L-ornithine 5-monooxygenase and Lon protease were down-regulated (1.46 and 1.25-fold, respectively) in the starved planktonic cells.

### Biofilm dispersal is an energy-requiring process

**Figure 3:**
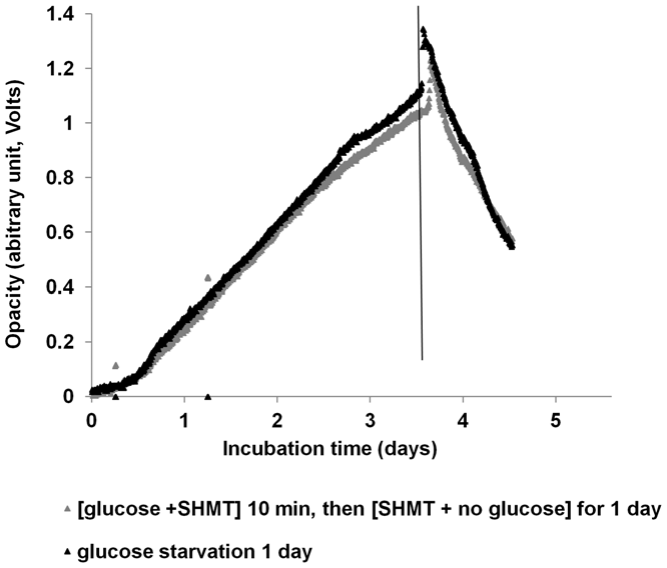
Effect of stringent response on biofilm dispersal. Effect of 200 µg ml^−1^ SHMT on biofilms of *P. aeruginosa* PAO1 WT, grown under continuous flow conditions in M9 medium (100 µM CaCl_2_) was assessed. The biofilms were exposed to glucose starvation in the presence (light grey) and absence (black) of SHMT. The experiments were repeated twice on different days. Data shown are representative biofilm-opacity measurements and are from one experiment. Glucose starvation was induced at the time indicated by the vertical line.

**Figure 4:**
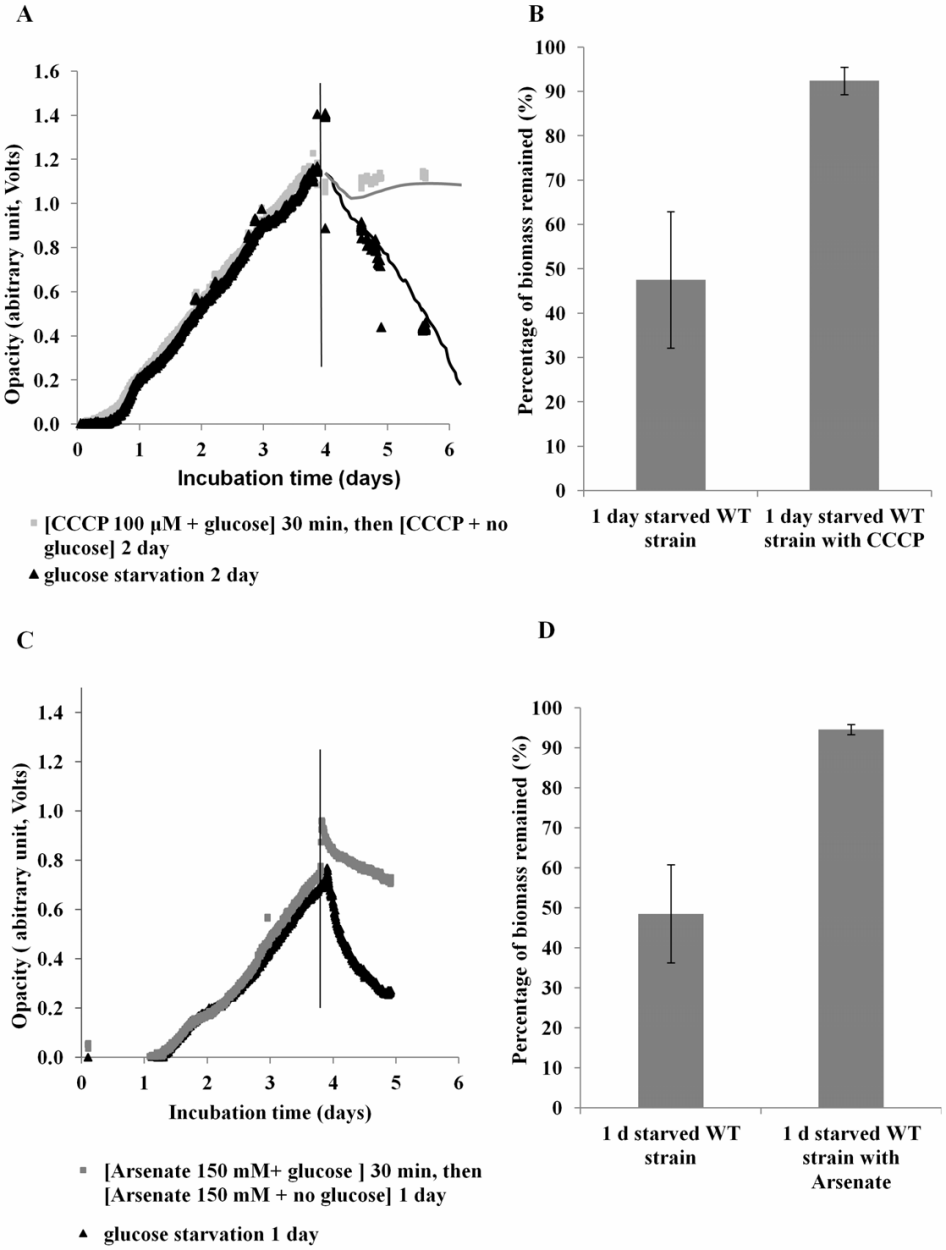
Effect of 100 µM CCCP and 150 mM arsenate on biofilms of *P. aeruginosa* PAO1 WT during glucose starvation. Biofilms grown under continuous flow conditions in M9 medium (100 µM CaCl_2_) were exposed to glucose starvation in the presence (light grey) and absence (black) of (A) CCCP or arsenate (C). Glucose starvation was induced at the time indicated by the vertical line. (B) Comparison of biomass remaining after 1 day of glucose starvation with and without CCCP as determined by the biofilm-opacity monitoring system. Error bars represent standard error (n = 3).

Biofilms were treated with L-serine hydroxamate (SHMT) or carbonyl cyanide *m*-chlorophenylhydrazone (CCCP) under starvation conditions to determine if the stringent response or the proton-motive force are involved in starvation-induced dispersal. In addition, starved biofilms were treated with arsenate to determine if dispersal requires ATP synthesis. SHMT treatment did not alter the dispersal response when observed by biofilm-opacity measurement (Fig. 3). However, while glucose-starved biofilms of *P. aeruginosa* PAO1 treated with CCCP showed an initial decrease in biofilm density from an OD of 1.15 to 1.05 (Fig. 4A), the biofilm did not disperse further. In contrast, the untreated, glucose-starved biofilm decreased in OD from 1.11 to 0.43 over the 2-day period (Fig. 4A), suggesting that proton-motive force is essential for the dispersal process (Fig. 4A). On average, after 1 day of glucose starvation, the CCCP-treated biofilms grown in the biofilm-opacity monitoring system showed a reduction of only 8% of the biofilm biomass, while the non-CCCP-treated biofilms were reduced by 53% (t test, *p*<0.05) (Fig. 4B). To determine if ATP synthesis is necessary for dispersal, the biofilm was treated with 150 mM arsenate and, as can been seen in Figure 4C, dispersal was inhibited in a manner similar to the CCCP-treated cells. Therefore, biofilm dispersal requires energy from ATP by direct synthesis (substrate-level phosphorylation) and through proton-motive force (oxidative phosphorylation).

### Role of *cyaA* in the starvation-induced dispersal of *P. aeruginosa*

**Figure 5:**
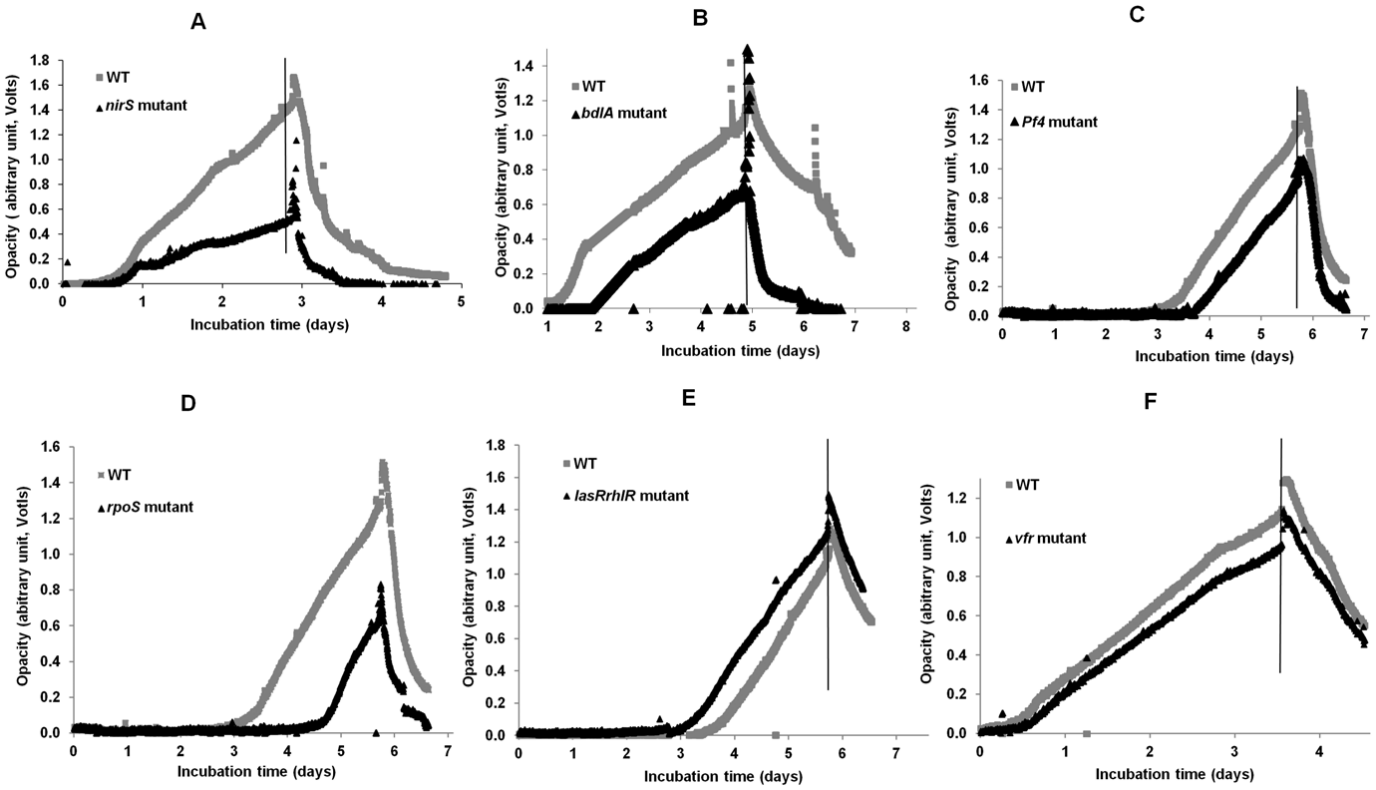
Biofilm formation and response to glucose starvation of *P. aeruginosa* PAO1 WT and mutant strains. Strains mutated in (A) *nirS*, (B) *bdlA*, (C) Pf4 phage, (D) *lasRrhlR*, (E) *rpoS* and (F) *vfr* were grown under continuous flow conditions in M9 medium (100 µM CaCl_2_). Glucose starvation was induced at the time indicated by the vertical line. Data represent single experiments.

The results presented above confirm that starvation-induced dispersal is an active process. Furthermore, the proteomic data suggests that starvation-induced dispersal results in subtle changes to the proteome. Therefore, strains that carried mutations in pathways involved in either biofilm dispersal (*nirS*, bacteriophage Pf4, *bdlA*, and quorum sensing), the starvation response (e.g. *rpoS* or *vfr*) or metabolism (e.g. *cyaA*) were investigated to determine if these loss of function mutants were altered in their starvation-induced dispersal response. Biofilms of the various mutant strains were formed in the on-line monitor and the starvation-induced dispersal response compared to the isogenic WT. The experiments were conducted three times on different days. All of the mutants, except for *vfr* and *rpoS*, showed a delay in biofilm formation (Fig. 5A–F); however, all of the mutants showed dispersal patterns similar to the WT. Statistical analysis revealed that the difference in percentage of biomass remaining for the WT and mutant strains at the end of the experiment was not significant (t test, *p*>0.05), *i.e.* the WT and mutant strains dispersed similarly. This suggests that these genes (encoding the nitric oxide-related dispersal protein BdlA, the general stress response sigma factor RpoS, the filamentous bacteriophage Pf4, quorum sensing and the receptor of the second messenger cyclic AMP synthase, Vfr) are not essential for the starvation-induced dispersal pathway.

**Figure 6:**
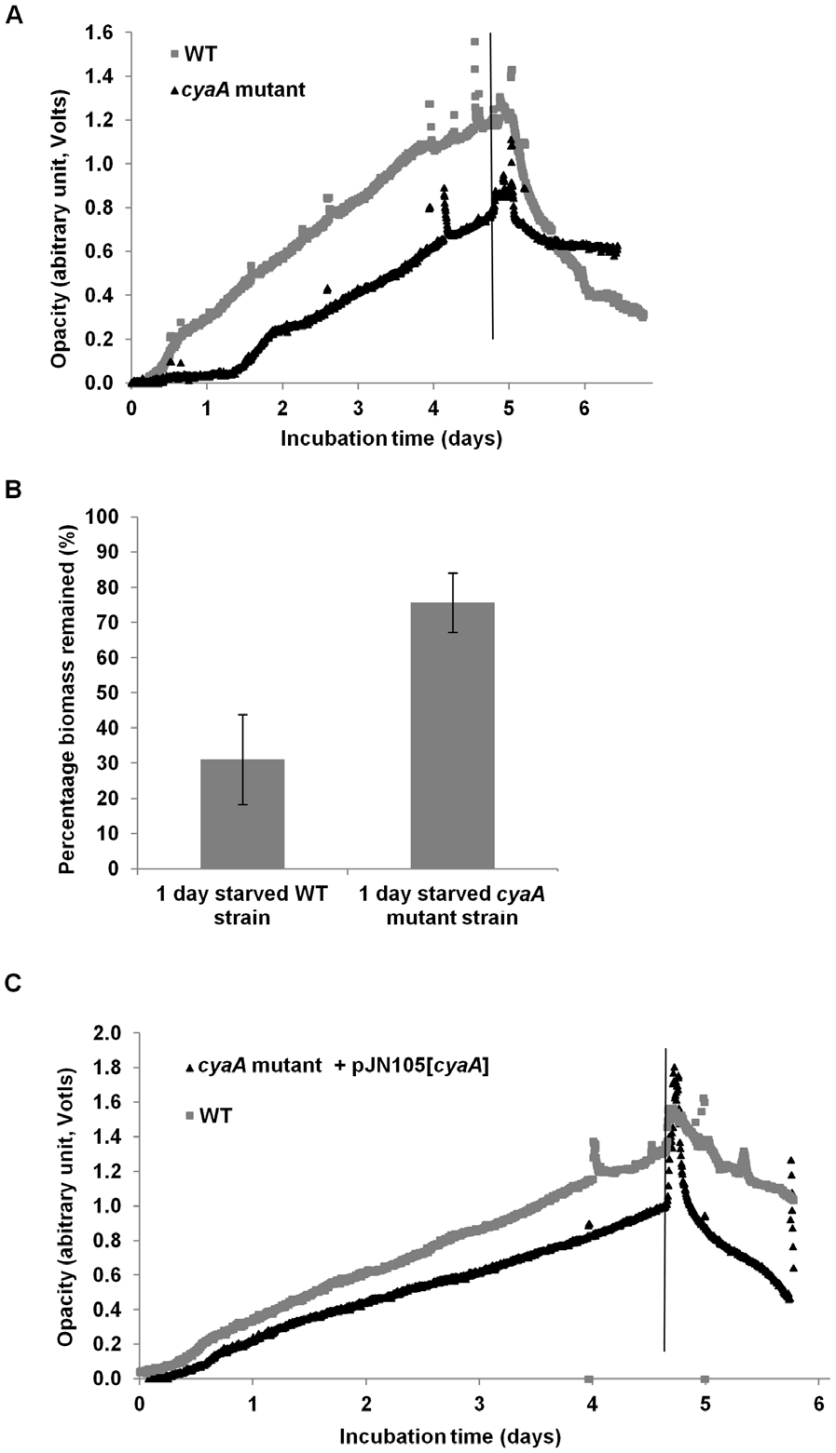
Biofilm formation and response to glucose starvation. *P. aeruginosa* PAO1 WT (grey) and *cyaA* mutant (black) biofilm formation and response to starvation was determined by biofilm-opacity measurement (A). Percentage of of *cyaA* biomass remaining after 1 day of glucose starvation (*p*<0.05) as determined by the biofilm-opacity monitoring system (B); error bars represent standard error. Biofilm formation and response to glucose starvation of *P. aeruginosa* PAO1 WT (grey) and complemented *cyaA* mutant (black) as determined by biofilm-opacity measurement (C). Glucose starvation was induced at the time shown by the vertical line.

A mutant in the *cyaA* gene was generated in *P. aeruginosa* PAO1 and the dispersal response was compared to its isogenic WT. Upon glucose starvation, the *cyaA* mutant showed an initial decrease in biofilm density (OD 0.75 to 0.63), but after 16 h, did not decrease any further as the OD remained constant (OD = 0.63) for 2 days (Fig. 6A). In contrast, the WT was observed to disperse rapidly, with the OD decreasing from 1.20 to 0.82 within 16 h, and to 0.36 after 2 days (Fig. 6A). After 1 day of starvation, the *cyaA* mutant biofilm was reduced by 24% compared to 69% for the WT biofilms (Fig. 6B). Statistical analysis (t test) revealed that the difference in biomass remaining for the WT and *cyaA* strains was significant (*p*<0.05). Complementation of the *cyaA* mutant, by reintroduction of the gene on an arabinose inducible plasmid, pJN105, restored the WT dispersal phenotype (Fig. 6C).

### The role of cAMP in starvation-induced biofilm dispersal

**Figure 7:**
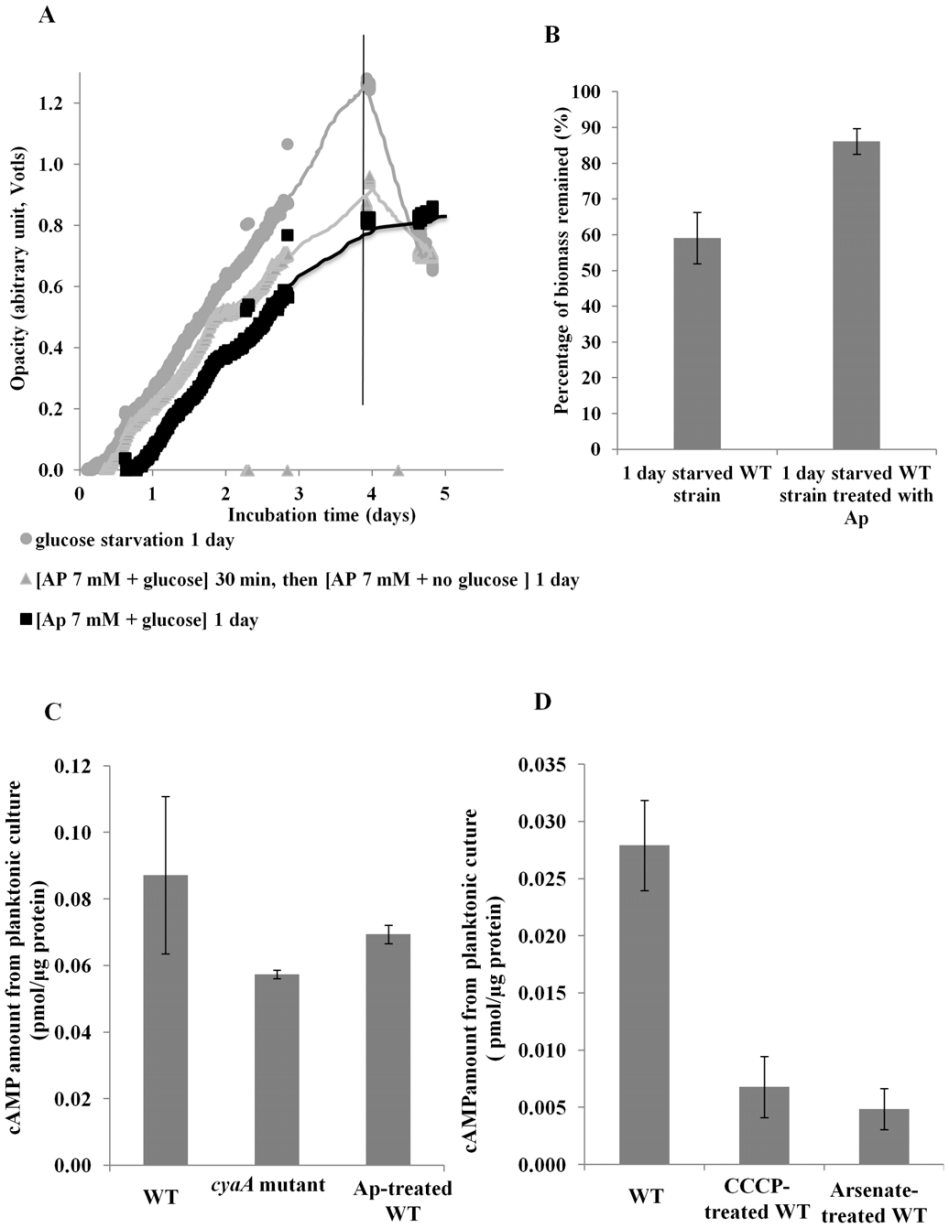
Effect of the inhibition of cAMP synthesis on the dispersal of PAO1 WT biofilms. The effect of inhibition of cAMP synthesis on dispersal of biofilms grown under continuous flow conditions in M9 medium (100 µM CaCl_2_) or M9 medium containing 7 mM atropine (Ap) as assessed photometrically (A) and percentage of biomass remaining after 1 day of glucose starvation with and without 7 mM Ap (*p*<0.05) (B) were determined. Intracellular cAMP levels in WT, *cyaA* mutant and Ap-treated *P. aeruginosa* planktonic cells were determined (C). Effect of CCCP and arsenate treatment on intracellular levels of cAMP in *P. aeruginosa* grown in planktonic culture (D). Two sets of samples were prepared on different days, and were measured in triplicate (n = 6). Error bars indicate standard error.

**T**he addition of 7 mM atropine (Ap) reduced WT biofilm dispersal compared to the untreated biofilms (Fig. 7A). The addition of Ap in the presence of glucose (non-starved) reduced, but did not completely inhibit, further biofilm formation. After 1 day of starvation, the Ap-treated biofilm was reduced by 14% compared to 44% for those not treated with Ap (Fig. 7B presents the results of two independent experiments). Statistical analysis showed that the difference in percentage of biomass remaining for the Ap-treated and non-treated biofilms was significant (*p*<0.05). To determine if the effect on dispersal was related to cAMP levels, intracellular cAMP concentrations of planktonic cultures of the *cyaA* mutant strain and Ap-treated cells were determined. Results revealed that the *cyaA* mutant produced less cAMP than the WT (0.057 and 0.087 pmol µg^−1^ protein respectively, Fig. 7C). The amount of cAMP in the Ap-treated WT was slightly higher than in the *cyaA* mutant (0.087, 0.069, respectively Fig. 7C) but was significantly lower than the untreated WT control (0.065 vs 0.087 respectively). Statistical analysis showed that the decreased cAMP levels in the *cyaA* mutant and Ap-treated WT were statistically significant (*p*<0.05). Furthermore, we assessed the intracellular cAMP levels of CCCP- and arsenate-treated cells and results showed that these cells also had a lower cAMP level compared to the untreated WT (Fig. 7D)

## Discussion

### Dispersal of *P. aeruginosa* WT biofilms under glucose starvation

Dispersal, which represents the completion of the biofilm life-cycle, is suggested to be important for the escape of cells from the biofilm when resources become limiting [11], [15], [17]. There is growing evidence that dispersal is an active process, where bacteria respond to a variety of signals (e.g. AHLs, diffusible signal factor) and environmental cues (e.g. nutrient concentration) which are perceived by the cell and transduced through an intracellular regulatory cascade (such as c-di-GMP) that represses biofilm related genes and upregulates planktonic phenotypes (e.g. motility) [12], [27], [28]. In order to gain further insights into the mechanisms by which *P. aeruginosa* biofilms disperse under carbon starvation, we first established continuous biofilm-opacity monitoring (OD_580 nm_ determination) (cf. ref. [15]) in a traditional flow-cell setup to facilitate quantification of biofilm dispersal. Dispersal in the biofilm-opacity monitoring system was compared to that of biofilms grown in traditional continuous flow cells, and it was determined that biofilms formed and dispersed similarly in both systems in response to glucose starvation. This study confirms previous reports that glucose starvation induces a very rapid dispersal response in *P. aeruginosa* PAO1 biofilms [14], [15], which, as determined in our growth system, occurs within 5 min of glucose depletion (Fig. 1C). On average, about half of the total biofilm biomass had dispersed 1 day after starvation in our growth system (Fig. 1AB). Furthermore, the dynamics of biofilm dispersal (Fig. 1C) showed that the loss of biomass was maximal at the beginning of the dispersal event and decreased thereafter.

The changes in protein synthesis were quantified for the starved and unstarved biofilm and dispersal cell populations as observed in our continuous flow system (cf. Fig. 1C), in order to identify those proteins involved in the carbon-starvation and dispersal responses. More than 100 proteins from a variety of functional groups were differentially expressed between the biofilm and dispersal cells during glucose starvation. This suggests that starvation and dispersal are complex processes and involve a general remodeling of cell physiology to prepare the biofilm cells for starvation and planktonic survival. While the response was broad and differences were typically not more than 2-fold, there were patterns of protein synthesis that correspond to the change from a sessile to a motile life-style. For example, proteins involved in motility were upregulated during glucose starvation-induced dispersal in both starved planktonic (PilQ, PilM) and biofilm (FimV, PilA, FliC) cells, which is consistent with previous observations of dispersal cells [12], [29]. Flagella-related motility is required in the initial attachment phase of biofilm development, however, after irreversible attachment flagella are no longer required and are absent in biofilm cells [29]. In contrast, PilA was detected in biofilms after attachment and throughout 7 days of biofilm development, but was not detected in the planktonic cells [30], while FliC expression was increased in the starved biofilm cells of *P. aeruginosa*. These observations support the suggestion that there was a switch from flagellum-based motility to swarming or twitching motility in the biofilm [30], whereas, in the last stage of the biofilm life cycle when biofilm cells disperse and revert to the planktonic life-style, flagella motility was required for active dispersal [29], [31]. Interestingly, motile cells have been observed in the center of cell clusters or microcolonies which were surrounded by non-motile cells [29] suggesting that increased motility is essential for dispersal and is induced prior to evacuation of bacteria from the biofilm.

Proteomic analysis also showed that proteins involved in carbon metabolism, energy generation, transcription and translation, biosynthesis of cofactors and prosthetic groups, and membrane transport, were differentially expressed upon starvation [29]. The glycolysis specific enzyme glyceraldehyde-3-phosphate dehydrogenase, was down-regulated in starved biofilm cells, suggesting that carbon metabolism was repressed in biofilm cells during glucose starvation. Further, differentially expressed proteins involved in energy generation in starved vs. non-starved cells included tricarboxylic acid (TCA) cycle proteins such as succinate dehydrogenase, which have also been shown to be expressed at higher levels in mature and late-stage biofilms compared to early-stage biofilms [29]. In contrast, Whiteley *et al.* (2001) revealed that these enzymes were 4-fold down-regulated in a 5 day-old *P. aeruginosa* biofilm [32]. It is possible that these 5 day-old biofilms were not at the dispersal stage of the biofilm life cycle or contained only small numbers of dispersed cells. In addition, some outer membrane proteins were differentially expressed during starvation in planktonic cells, including the upregulation of OprG, porin F and peptidoglycan-associated lipoprotein, while proteins such as the insulin-cleaving metalloproteinase, were down-regulated indicating that changes in cell membrane structure or hydrophobicity may be involved in the dispersal process. The low (generally 2-fold or less) overall change in protein synthesis may either be a reflection of the overall heterogeneity of the biofilm (where protein changes represent an average across the different parts of the biofilm) or may indicate that starvation-induced dispersal is a consequence of global changes in physiology to prepare the cells to convert from a sessile life-style to a free living planktonic life style.

To demonstrate that starvation-induced dispersal is in fact a regulated, active process, we investigated the effect of inhibitors of cell metabolism on the dispersal response. Preliminary evidence indicated that biofilms treated with the protein synthesis inhibitor, chloramphenicol, dispersed in a similar fashion to the untreated biofilm, suggesting that protein synthesis is not required for glucose starvation-induced dispersal (data not shown). This data was further supported by the fact that SHMT treatment also failed to inhibit dispersal (Fig. 3) indicating that the stringent response was not required for starvation-induced dispersal. This may also suggest why overall changes in protein levels were low in the dispersing populations. In contrast, *P. aeruginosa* biofilms were defective for dispersal when energy generation was inhibited by addition of the proton-ionophore CCCP or arsenate. CCCP is a proton uncoupler that disrupts the proton-motive force and consequently inhibits ATP synthesis [33]–[35], whereas arsenate is a phosphate analog that competes with inorganic phosphate also in the phosphate-level phosphorylation of metabolic pathways, e.g. in glycolysis, hence inhibiting ATP synthesis completely [36]. These data clearly indicate that starvation-induced dispersal is not a passive process, but is active and requires ATP. For example, energy is important for microbial motility [29], an important mechanism used by biofilm cells to aid in the escape of cell from the biofilm [12]. It is also possible that energy is required for enzymatic cleavage of EPS matrix components, which has been shown to be important for dispersal [37], [38]. The energy dependence may also be directly linked to the cAMP pathway since cAMP is synthesized from ATP [39]. This is supported by the arsenate treatment which inhibited dispersal (Fig. 4C) and which was directly shown to reduce intracellular levels of cAMP (Fig. 7D).

To identify specific effectors or regulators that control starvation-induced dispersal, a selection of mutants were tested which were selected from previous studies on biofilm development, or that were linked to starvation and stress adaptation. Mutations in *nirS*, *bdlA*, *rpoS*, *vfr*, bacteriophage Pf4, and AHL-mediated quorum sensing had no effect on the dispersal response. The quorum sensing double mutant and the *rpoS* mutant formed less biofilm biomass compared to the WT, indicating that quorum sensing mediates biofilm development [40]. Nitric oxide, produced by *nirS*, has been shown to mediate dispersal via c-di-GMP [20], as has starvation-mediated dispersal [15]. Despite operating via the same intracellular second messenger, dispersal of the *nirS* mutant was no different from the wild-type during starvation, which suggests that NO and starvation control dispersal by different pathways. Previously it was reported that *nirS* biofilms grow faster than the isogenic WT in a continuous flow cell system [196]. However, in our growth system, the *nirS* biofilms grew much slower than the WT, which can be due to differences in the surfaces that the biofilms are attaching to or to other differences in the way the two systems were run. In our growth system, most of the mutants, including *nirS*, grew slower and reached less final biomass than did the WT strain.

In contrast to the mutants described above, a *cyaA* mutant failed to disperse upon carbon starvation. To the authors knowledge, this is the first demonstration of a role of cAMP in the response of *P. aeruginosa* PAO1 WT biofilms to glucose starvation. The second messenger cAMP plays a major role in the regulation of metabolism repression in *E. coli*, and in addition, is a major regulator of virulence gene expression in *P. aeruginosa* [41], [42]. The results described above showed that a *cyaA* mutant formed less biofilm than its isogenic WT and exhibited dispersal for the first few hours, after which there was no further dispersal, indicating that cAMP is in some way involved in the dispersal mediated by glucose starvation. The complementation of *cyaA* restored the biofilm dispersal phenotype (Fig. 6). The role of cAMP in dispersal was also supported by the addition of atropine, which inhibited dispersal and was shown to reduce cAMP in planktonic cultures (Fig. 7C). Unlike cAMP, Vfr, which is the cAMP receptor protein in *P. aeruginosa*, does not seem to control dispersal, as the *vfr* mutant biofilms behaved similarly to the WT under glucose depletion (Fig. 5F). *P. aeruginosa* encodes a second cAMP binding protein, CbpA, which is not involved in Vfr mediated phenotypes such as virulence factor expression [43] and currently no phenotype has been ascribed to this protein. It is tempting to speculate that, unlike Vfr, this protein may play a role in biofilm dispersal. In *Enterobacteriaceae*, cAMP is produced by a single adenylate cyclase [39]; however, in *P. aeruginosa* cAMP is produced by two adenylate cyclases, encoded by *cyaA*, a Class I cAMP synthase, and *cyaB*, a Class III cAMP synthase that contains putative transmembrane domains [39]. The majority of the cellular cAMP is produced by CyaB rather than by CyaA [42], [44] and it is possible that a *cyaB* mutant will be more severely impaired in the dispersal response under nutrient limitation.

We similarly tested a *lapG* mutant and observed that it was also defective in starvation-induced biofilm dispersal (data not shown). LapG is a membrane bound protease that, in a c-di-GMP dependent fashion [17], cleaves the surface adhesion LapA in *Pseudomonas putida*, to mediate dispersal. Starvation of *P. aeruginosa* was previously linked to a decrease in c-di-GMP during dispersal [15] and it remains to be determined if cAMP and c-di-GMP operate through the same pathway involving Lap or through parallel pathways in order to mediate dispersal.

In conclusion, our study shows for the first time that starvation-induced dispersal in *P. aeruginosa* operates through the intracellular second messenger cAMP and that dispersal is an active process, requiring a membrane potential and energy at the expense of proton-motive force. In addition, the starvation-induced dispersal pathway appears to operate independently from the NO-mediated dispersal pathway, despite the observation that both NO exposure and starvation lead to reduced levels of c-di-GMP. Proteomic analysis of proteins involved in dispersal during glucose starvation indicated that the starvation and dispersal response is a sophisticated program that involves many receptor, effector and regulatory proteins.

## Materials and Methods

### Bacterial strains and growth conditions

Bacterial strains were routinely grown in Luria-Bertani (LB) broth [15], [21] or on LB plates containing 1.5% agar. The bacterial strains and plasmids used in this study are listed in Table 1. Strains were maintained at −80°C. Prior to each experiment, cultures were inoculated from freshly-streaked plates and grown overnight in M9 [15] medium supplemented with 10 mM glucose. For cultivation of *P. aeruginosa* strains, antibiotics were used where necessary at the following concentrations, gentamycin (Gm) 150 µg ml^−1^, tetracycline (Tc) 50 µg ml^−1^; for *E. coli* strains, ampicillin (Ap) 50 µg ml^−1^, Gm 30 µg ml^−1^ and Tc 10 µg ml^−1^.

**Table 1:** Strains, plasmids and primers used in this work.

Strains	Relevant characteristics	Source or reference
*Pseudomonas aeruginosa*		
PAO1 MA67	Wild-type PAO1, MA67	[15]
PAO1 UWGC	Wild-type PAO1	UWGC [52]
PAO1	Wild-type PAO1, ATCC	[53]
PAO1UWGC *cyaA*	PAO1-UWGC; Strain ID 9864, Tc^r^	UWGC [52]
PAO1 MA67 *cyaA*	PAO1 MA67; *cyaA*, Tc^r^	This study
PAO1 MA67 *cyaA*:pJN105[*cyaA*]	PAO1 MA67 *cyaA* carrying pJN105[*cyaA*]	This study
PAO1 Δ*lasRrhlR*	PAO1 ATCC, Δ*lasR-rhlR*	[53]
PAO1 ΔPf4	PAO1, Δ(Pf4), Gm^r^	[19]
PAO1 *rpoS*	Strain ID 132, *rpoS*, Tc^r^	UWGC [52]
PAO1 *vfr*	PAO1 with Tc cassette inserted into *Bcl*I site of *vfr*, Tc^r^	[53]
PAO1 *bdlA*	PAO1; *bdlA*::pKO[*bdlA*]	[21]
*Escherichia coli*		
DH5-α	F_ 80*lac*ZDM15 D(*lac*ZYA-*argF*) U169 *recA*1 *endA*1 *hsdR*17(rk_, mk+) *phoA supE*44 *thi*-1 *gyrA*96 *relA*1 *tonA*	[54]
**Plasmids**		
pJN105	pJN105 *araC*-PBAD cassette cloned in pBBR1MCS-5, Gm^r^	[48]
pJN105[*cyaA*]	pJN105 inducible vector with fragment (2.853 kb) of *cyaA*	This study
**Primers**		
cyaA_F	5′- gcttccgggcgatacaatgg -3′	This study
cyaA_R	5′- cggcgccagcgagcagggtaatac -3′	This study

Abbreviations: Tc^r^ = tetracycline resistance; Gm^r^ = gentamicin resistance, UWGC = University of Washington Genome Center, ATCC = American type culture collection.

For biofilm experiments, cells from overnight cultures (shaken at 200 rpm, 37°C) were collected by centrifugation at 6000× *g* for 5 min, resuspended in fresh medium, incubated for 30 min at 37°C with shaking (200 rpm), and subsequently used for inoculation into the continuous flow cell systems (see below).

### Biofilms pre-grown and starved in standard continuous flow cells for CSLM

Biofilms were grown in continuous flow chambers for CLSM as described previously [19] with some modifications. The sterile flow cells were connected to a sterile feed and waste collection bottle with sterile oxygen-permeable silicon tubing (Silastic laboratory tubing sizes 10 and 15; Dow Corning Corporation). M9-salts medium [15] supplemented with 10 mM glucose and 100 µM CaCl_2_ was fed at a rate of 9 ml min^−1^ through the system using a peristaltic pump (Watson-Marlow Bredel, Sci-Q 323S). Cells from overnight cultures of *P. aeruginosa* strains (see above) were inoculated into each flow cell and incubated without flow at room temperature for 1 h to allow cell attachment. The biofilms (in duplicate) were incubated for 4 days and experiments were repeated three times on different days. Glucose starvation was applied to 4 day-old biofilms by switching from the glucose/M9 feed solution to M9 salts-medium lacking a carbon source. Starvation was applied for either 5 or 24 h, before biofilms were stained (Live/Dead) and analyzed by CLSM (see below).

### Biofilm visualization and quantification using CLSM

The biofilms grown in flow cells (polycarbonate body covered with glass slide as a substratum for biofilm proliferation, channel dimensions,1×1×40 mm) [19] were stained with the Live/Dead *BacLight* bacterial viability kit (Molecular Probes Inc., Eugene, OR, USA) in the dark for 20 min at room temperature. The stained biofilm was analyzed using CLSM (Olympus FV1000, Olympus Optical Co. Ltd, Tokyo, Japan). Five images were recorded randomly along the flow cells at different locations and Image J software (version 1.36b (http://rsb.info.nih.gov/ij/)) was used to quantify biomass and surface coverage.

### Biofilms pre-grown and starved in modified continuous flow cells designed for photometrical biofilm-density monitoring

The standard continuous-flow setup (above) was modified to follow the dynamics of biofilm dispersal photometrically, as the decrease of turbidity (OD_580 nm_)/opacity of the cellular biofilm biomass during starvation, firstly, by replacing the standard flow cells with similar-sized glass tubes (inner-diameter 2.8 mm, 6.0 cm in length) for growth of the biofilms. The glass tubes were connected to the medium flow, and inoculated and incubated, as described above. Secondly, an LED (580 nm+/−10 nm) on one side of the glass tube, and a photosensor (peak sensitivity 600 nm) on the opposite side of the glass tube, was set up to monitor the turbidity/opacity through the glass tube; the light path was restricted to a 2 mm diameter section of biofilm located 3 cm behind the start of the glass tube/inoculation point of the biofilm. A set of biofilm-photometers was constructed by the workshop of the University of Konstanz [15], [45]; the photometer signals were computerized using a datalogger (Labjack U12, Meilhaus, Germany) and visualized on a computer (DAQfactory-express software, Azeotech, USA). Opacity/turbidity was recorded every 5 min. Cultures of *P. aeruginosa* PAO1 WT or mutants (*nirS*, Pf4, *bdlA, rpoS*, *lasRrhlR*, *vfr* and *cyaA*) were inoculated separately into the glass tubes and grown at room temperature for 3 to 5 days before the feed medium was switched to the starvation medium (see above). Effluents from the biofilms were collected for assessment of colony forming units (CFU), photometric quantification of OD_580 nm_, and glucose concentration determination (see below). The experiments for the WT, *nirS*, *bdlA*, *Pf4*, *lasRrhlR*, *rpoS*, and *vfr* mutant strains were repeated three times on different days, and the experiments for the *cyaA* mutant were repeated three times in duplicate.

### Determination of glucose concentration and optical density of biofilm effluents

Glucose concentrations were measured in biofilm effluents using a glucose assay kit (GAGO20-1KT, Sigma-Aldrich Inc., Castle Hill, Australia). In order to measure the biomass of the biofilm effluent, the OD of the effluent was determined once per day and every hour for 5 h after the glucose starvation was induced.

### Quantification of biofilm effluent biomass and CFU

The dispersal cell biomass was quantified by measuring the OD of the biofilm effluent once per day over the incubation time. The OD was determined at 580 nm using a spectrophotometer (Pharmacia Biotech, Novaspec II). In order to determine the number of culturable cells that dispersed from the biofilm, CFUs were assessed by the spread plate method once per day or every hour for biofilms starved for 5 h after glucose starvation was induced. For this, serial dilutions were performed using PBS as the diluent and 20 µl of the diluted sample was spread on LB agar plates and the plates incubated at 37°C for 24 h.

### Cultivation of biofilms for harvesting and preparation of protein extracts

To generate the protein samples for iTRAQ proteomic analysis (see below), the biofilms were pre-grown and starved in the continuous-flow biofilm-photometer setup as described above with the exception that the biofilm biomass was harvested from the silicon tubing that connected to the waste-collection bottle (20 cm, Silastic Laboratory tubing, size 10, ID 1.5 mm) in order to provide sufficient biomass for analysis. The biofilms were grown for 4 days before glucose starvation was applied, and harvested 2 h after starvation. One biofilm served as a control (unstarved). Cellular biomass in the effluent was collected for 2 h (starved and unstarved control) to ensure that enough biomass was obtained for protein analysis. Briefly, effluents were collected into sodium azide (0.01%), added in portions to the collected effluent, and the mixture was placed on ice. After harvesting, the cells were collected by centrifugation at 12,000× *g* for 10 min at 4°C.

Proteins were extracted and prepared as previously described [29] with some modifications. The cell pellets were resuspended in 200 µl lysis buffer (10 mM sodium phosphate pH 8, 10 mM EDTA pH 8, 0.05% SDS) containing 0.3 mg ml^−1^ of phenylmethylsulfonyl fluoride. Cells were ruptured by sonication (on ice) using a digital sonifier (Branson, USA) at 30% amplitude, 5 times 30 s bursts with 0.5 s for each on/off interval. The cell debris and unbroken cells were removed by centrifugation at 25,000× *g* for 10 min at 4°C. The supernatant containing cellular proteins were stored at −80°C until analysis. Total protein concentrations of the samples were determined by use of the BCA protein assay using bovine serum albumin as a standard.

### iTRAQ proteomic analysis

Protein samples (approximately 50–100 µg each) were reduced, digested and labeled with iTRAQ reagents followed the manufacturer's recommendations (The iTRAQ® Reagents Application Kit, Applied Biosystem). Samples were pooled, fractionated and analyzed by tandem mass spectrometry (MS/MS). A Mascot database search was performed to identify the labeled peptides and proteins using the ProQuant program of ProGroup software package (2.0.1 version, Applied Biosystems) for data analysis. Samples were pooled and fractionated and analyzed by tandem mass spectrometry (MS/MS). A Mascot database search was performed to identify the labelled peptides and proteins using the ProQuant 1.0 program and ProGroup software (2.0.1 version, Applied Biosystems) for data analysis. The ProGroup software was applied to ensure that each MS spectrum is used to support the identification of only one protein. ProGroup reports were produced for further quantitation analysis with protein confidence level set at 95% confidence. Two independent experiments were conducted on different days.

### Induction of stringent response

To induce the stringent response via amino acid starvation, 200 µg ml^−1^ SHMT [46] (dissolved in MQ water) was added to 4 day-old WT biofilms. SHMT was added to the glucose- containing medium for 10 min before the onset of glucose starvation and during glucose starvation. The control biofilms were starved for glucose without SHMT addition.

### Inhibition of energy production

CCCP [47] and arsenate (sodium arsenate dibasic heptahydrate) [36] were used to inhibit energy production of PAO1 biofilm cells grown in the online biofilm monitoring system for 4 d before glucose starvation was applied. The biofilms were pre-treated with 100 µM CCCP (dissolved in 0.1% DMSO) or 150 mM arsenate (dissolved in MiliQ water) for 30 min, followed by switching from glucose-containing feed to M9 lacking glucose in the presence of CCCP or arsenate. Two independent arsenate experiments were carried out in duplicate on different days. For CCCP experiments, control biofilms were treated with DMSO alone or with CCCP and DMSO in the presence of glucose. During the treatment, the whole biofilm setup was wrapped in aluminum foil as CCCP is sensitive to light. Furthermore, the LEDs of the biofilm-photometers were turned on for only 1 min every 1 or 2 h, so that measurements could be made. Three independent CCCP treatments were conducted on different days.

### Generation of a *cyaA* mutant of *P. aeruginosa* PAO1 MA67

A *cyaA* mutant of *P. aeruginosa* MA67 WT was generated by phage transduction as previously described (33) with some modifications. The *P. aeruginosa cyaA* mutant donor (from University of Washington Genome Center) and WT phage (approximately 100 PFU ml^−1^) were used. The transducing phage (about 5×10^8^ PFU ml^−1^) was attenuated by UV treatment at 50 J m^2−1^ (Ultraviolet Crosslinker, Amersham Life Science). For transduction, 500 µl of an overnight culture of the recipient strain was centrifuged for 3 min 20,000× *g* and resuspended in 500 µl of TNM buffer (10 mM Tris HCl [pH 7.4], 150 mM NaCl, 10 mM MgSO4), mixed with 500 µl of attenuated transducing phage and incubated at 37°C for 15 min to allow phage absorption. Non-adsorbed phage were removed by washing twice in TNM buffer followed by centrifugation for 3 min at 20,000× *g*. The pellets were resuspended in 0.5 ml TNM, plated onto selective media (LB10 with 50 µg ml^−1^ Tc) and incubated at 30°C for 2 to 3 days until the transduced colonies appeared.

### Complementation of the *cyaA* mutant strain

The *cyaA* gene (2.853 Kbp; PA5272) was PCR amplified from 20 ng of genomic DNA of *P. aeruginosa* PAO1, using *Pwo* polymerase and the following primers: forward (5′- GCT TCC GGG CGA TAC AAT GG -3′) and reverse (5′- CGG CGC CAG CGA GCA GGG TAA TAC -3′). The PCR product was extracted using a gel purification kit following the manufacturer's instructions (Invitrogen, Australia) and eluted with 43 µl molecular grade water (Eppendorf, Australia). Subsequently, 38 µl of purified PCR product were used in an appropriate reaction mixture with *Taq* polymerase for 20 min at 72°C in order to generate A-overhangs. The products were purified with a PCR purification kit (Invitrogen, Australia) and eluted in 30 µl molecular grade water.

The *cyaA* gene was cloned into the pCR4-TOPO vector according to the manufacturer's instructions (Invitrogen, Australia). After incubation, the reaction mixture was dialyzed for 20 min using 0.025 µm filters (MF, Millipore, Bedford, USA) to remove salts and subsequently transferred into electrocompetent DH5α *E. coli* cells by electroporation. After recovery of the cells at 37°C for 1 h, cells were subjected to Blue/White screening. Plasmids were extracted from white clones using the PureLink HQ Mini Plasmid purification kit (Invitrogen, Australia). The insert was confirmed by sequencing using M13F and M13R primers provided from the manufacturer. The PCR4-TOPO[*cyaA*] plasmid was then used for subcloning the *cyaA* gene, using the *Eco*RI restriction site, into the arabinose-inducible vector pJN105 [48] to create pJN105[*cyaA*]. After confirmation of the correct orientation of the gene, pJN105[*cyaA*] was used for transformation into electrocompetent *cyaA* mutant cells by electroporation.

Biofilms of the *P. aeruginosa* c*yaA* mutant carrying the pJN105[cyaA] and its isogenic MA67 PAO1 WT carrying the empty pJN105 vector were grown in the online monitor system as described below for 4 days. For the *P. aeruginosa cyaA* pJN105[*cyaA*] biofilms and for the WT carrying the empty pJN105 vector, Gm was added to the M9 medium at a final concentration of 10 µg ml^−1^ throughout the experiment. Arabinose (0.4%) was added at the same time that glucose starvation was applied to induce *cyaA* expression.

### Determination of intracellular cAMP levels

Intracellular levels of cAMP were determined from planktonic cultures of treated *P. aeruginosa* PAO1 WT (CCCP, 150 mM arsenate, atropine) *P. aeruginosa* PAO1 WT un-treated and an isogenic *cyaA* mutant (untreated). This was achieved by inoculating each strain, adjusted to an OD_600 nm_ of 0.1, into 5 ml of M9 supplemented with 10 mM glucose. After growth of the cultures at 37°C with shaking (200 rpm) to an OD_600 nm_ of 0.4, the cultures were incubated for another 2 h in the presence (*P. aeruginosa* PAO1) or absence (*P. aeruginosa* PAO1 WT and *cyaA* mutant) of either 100 µM CCCP, 150 mM arsenate or 7 mM atropine. Samples were prepared as per the manufacturer's recommendation with some modifications. Briefly, *P. aeruginosa* PAO1 *cyaA* mutant and WT cells were resuspended in 500 µl of 0.1 M HCl to inhibit phosphodiesterase (PDE) activity. One hundred µl aliquots of these samples were used for protein determination by the Lowry protein assay (see below). The remainder of the samples (400 µl) were incubated at room temperature for 20 min and then kept at −80°C until extraction. The samples were sonicated (Branson, USA) at 30% amplitude, four 30 s bursts with 0.5 seconds for each on/off interval (in ice). The cell debris and unbroken cells were removed by centrifugation at 25,000× *g* for 5 min at 4°C. Each sample was prepared in duplicate. The cAMP measurement assays were performed following the manufacturer's instructions (cAMP Direct Immunoassay Kit, BioVision, Mountain View, CA, USA). Two independent experiments were conducted on different days.

### Quantification of total protein

Total protein concentrations of the samples prepared for cAMP measurement were determined by the Lowry protein assay [49] with some modifications. Briefly, 250 µl of 3 M TCA were added to each protein sample to precipitate proteins. Bovine serum albumin (BSA) was used to generate protein concentration standards.

### cAMP inhibition with atropine

The *P. aeruginosa* PAO1 WT biofilms grown in the online system were treated with 7 mM atropine [50], [51] to reduce the intracellular cAMP levels. Atropine was added into the M9 medium of the 4 day-old biofilms. The control biofilms were treated with atropine in the presence of glucose. Two independent experiments were conducted on different days.

### Statistical analysis

IBM SPSS Statistics version 20.0.0 was used to perform Student's *t* tests. The significance level was set with 95% confidence. The significant differences in remaining/reduced biomass under control and experimental conditions were compared. The dataset size (n) for each experiment was indicated in each corresponding figure legend.

## Supporting Information

Table S1 **Differentially expressed proteins (118) with** ***p*** **-value<0.05 showing relative fold change from starved biofilm samples compared to non-starved biofilm samples.**
(DOCX)

Table S2 **Differentially expression proteins (109) with** ***p*** **-value<0.05 showing relative fold change from the starved effluent samples compared to the non-starved effluent samples.**
(DOCX)

Table S3 **Overlap of proteins identified in Tables S1 and S2 for biofilm and planktonic cells.**
(DOCX)
