# Supplementary material for: Glucose Starvation-Induced Dispersal of Pseudomonas aeruginosa Biofilms Is cAMP and Energy Dependent
Source: PLoS One. 2012 Aug 14;7(8):e42874. doi: 10.1371/journal.pone.0042874 (PMC3419228; doi:10.1371/journal.pone.0042874)
Supplement: Table S1 — Differentially expressed proteins (118) with p -value<0.05 showing relative fold change from starved biofilm samples compared to non-starved biofilm samples. (DOCX) [file pone.0042874.s001.docx]

**TABLE S1. Differentially expressed proteins (118) with *p*-value < 0.05 showing relative fold change from starved biofilm samples compared to non-starved biofilm samples**

| *N* | *Accession* | *Function* | *Name* | *Fold-change* | *P-Value* |
| --- | --- | --- | --- | --- | --- |
| 370 | Q9I194\|PVDQ_PSEAE | Adaptation, Protection | Acyl-homoserine lactone acylase pvdQ | 1.33 | 0.011 |
| 268 | Q9HWF9\|BFR_PSEAE | Adaptation, Protection | Bacterioferritin | -1.33 | 0.050 |
| 325 | Q9I168\|Q9I168_PSEAE | Adaptation, Protection | L-2,4-diaminobutyrate:2-ketoglutarate 4-aminot | 1.89 | 0.002 |
| 63 | Q51548\|PVDA_PSEAE | Adaptation, Protection | L-ornithine 5-monooxygenase PvdA | 1.35 | 0.003 |
| 132 | P95459\|CSPA_PSEAE | Adaptation, Protection | Major cold shock protein cspA | -1.64 | 0.000 |
| 82 | Q9I662\|Q9I662_PSEAE | Adaptation, Protection | Probable cold-shock protein | -1.39 | 0.013 |
| 185 | Q9I4H8\|Q9I4H8_PSEAE | Adaptation, Protection | Probable cold-shock protein | 1.26 | 0.044 |
| 350 | P57668\|TPX_PSEAE | Adaptation, Protection | Probable thiol peroxidase | -1.37 | 0.034 |
| 16 | Q9I157\|Q9I157_PSEAE | Adaptation, Protection | Pyoverdine synthase PvdL | 1.16 | 0.035 |
| 404 | Q9HU67\|Q9HU67_PSEAE | Adaptation, Protection | Regulatory protein TypA | 1.14 | 0.036 |
| 488 | Q51375\|LEU3_PSEAE | Amino acid biosynthesis and metabolism | 3-isopropylmalate dehydrogenase | 1.28 | 0.033 |
| 67 | Q9I685\|SAHH_PSEAE | Amino acid biosynthesis and metabolism | Adenosylhomocysteinase | 1.38 | 0.031 |
| 538 | Q9HUM6\|PURA_PSEAE | Amino acid biosynthesis and metabolism | Adenylosuccinate synthetase | 1.20 | 0.001 |
| 113 | P00282\|AZUR_PSEAE | Amino acid biosynthesis and metabolism | Azurin | -1.32 | 0.000 |
| 455 | Q9I2U6\|FOLD_PSEAE | Amino acid biosynthesis and metabolism | Bifunctional protein folD | 1.90 | 0.025 |
| 491 | Q9HUV9\|PUR9_PSEAE | Amino acid biosynthesis and metabolism | Bifunctional purine biosynthesis protein purH | 1.43 | 0.009 |
| 221 | O05926\|O05926_PSEAE | Amino acid biosynthesis and metabolism | CysB DNA-binding transcriptional dual regulator | 2.17 | 0.037 |
| 375 | Q9HW04\|ARGJ_PSEAE | Amino acid biosynthesis and metabolism | Glutamate N-acetyltransferase | 1.46 | 0.024 |
| 236 | Q9HYK7\|Q9HYK7_PSEAE | Biosynthesis of cofactors, prosthetic groups and carriers | Ferredoxin--NADP+ reductase | 1.41 | 0.000 |
| 219 | O68283\|ALKD_PSEAE | Carbon compound catabolism | 2-dehydro-3-deoxy-phosphogluconate aldolase | 1.14 | 0.044 |
| 176 | Q9HZK4\|Q9HZK4_PSEAE | Carbon compound catabolism | Probable glyceraldehyde-3-phosphate dehydrogen | -1.35 | 0.008 |
| 356 | Q9HU21\|Q9HU21_PSEAE | Cell wall / LPS / capsule | DTDP-4-dehydrorhamnose 3,5-epimerase | 1.39 | 0.031 |
| 205 | Q9X6V7\|Q9X6V7_PSEAE | Cell wall / LPS / capsule | Penicillin-binding protein 5 | -1.32 | 0.007 |
| 438 | Q9HWQ1\|Q9HWQ1_PSEAE | Central intermediary metabolism | Probable sulfite or nitrite reductase | -1.16 | 0.050 |
| 204 | Q9HVU0\|Q9HVU0_PSEAE | Cell wall / LPS / capsule | Rod shape-determining protein MreB | -1.39 | 0.036 |
| 220 | Q9X2N2\|6PGL_PSEAE | Central intermediary metabolism | 6-phosphogluconolactonase | -1.44 | 0.001 |
| 71 | Q9HUC3\|Q9HUC3_PSEAE | Central intermediary metabolism | Polyhydroxyalkanoate synthesis protein PhaF | 1.35 | 0.007 |
| 68 | Q9I6M5\|Q9I6M5_PSEAE | Central intermediary metabolism | Succinate-semialdehyde dehydrogenase | -1.37 | 0.045 |
| 2 | Q9HV43\|DNAK_PSEAE | DNA replication, recombination, modification and repair | Chaperone protein dnaK | 1.12 | 0.020 |
| 122 | Q9I7C4\|DPO3B_PSEAE | DNA replication, recombination, modification and repair | DNA polymerase III subunit beta | -1.41 | 0.047 |
| 130 | Q9I2W9\|Q9I2W9_PSEAE | Energy metabolism | Phosphoenolpyruvate synthase | -1.25 | 0.038 |
| 261 | Q9HVY6\|Q9HVY6_PSEAE | Energy metabolism | Probable cytochrome c1 | 1.75 | 0.000 |
| 323 | Q9I3D4\|Q9I3D4_PSEAE | Energy metabolism | Succinate dehydrogenase | 3.70 | 0.000 |
| 17 | P53593\|SUCC_PSEAE | Energy metabolism | Succinyl-CoA ligase [ADP-forming] subunit beta | -1.30 | 0.003 |
| 115 | O54439\|ACP1_PSEAE | Fatty acid and phospholipid metabolism | Acyl carrier protein 1 | -1.64 | 0.020 |
| 108 | Q9I4Z4\|PAL_PSEAE | Membrane proteins | Peptidoglycan-associated lipoprotein | 4.61 | 0.000 |
| 330 | Q9I183\|Q9I183_PSEAE | Membrane proteins | Pyoverdine biosynthesis protein PvdE | 4.10 | 0.011 |
| 23 | P72151\|FLICB_PSEAE | Motility & Attachment | B-type flagellin FliC | 1.35 | 0.000 |
| 15 | P04739\|FMPO_PSEAE | Motility & Attachment | Fimbrial protein PilA | 1.34 | 0.000 |
| 33 | Q9HZA6\|Q9HZA6_PSEAE | Motility & Attachment | Motility protein FimV | 1.37 | 0.004 |
| 444 | Q9HZ70\|KCY_PSEAE | Nucleotide biosynthesis and metabolism | Cytidylate kinase | 1.48 | 0.050 |
| 263 | O68822\|AMPA_PSEAE | Nucleotide biosynthesis and metabolism | Cytosol aminopeptidase | 1.60 | 0.005 |
| 333 | Q51551\|PYRX_PSEAE | Nucleotide biosynthesis and metabolism | Dihydroorotase-like protein | 1.19 | 0.026 |
| 163 | Q59636\|NDK_PSEAE | Nucleotide biosynthesis and metabolism | Nucleoside diphosphate kinase | 1.10 | 0.008 |
| 421 | Q9S646\|PPK_PSEAE | Nucleotide biosynthesis and metabolism | Polyphosphate kinase | 1.58 | 0.003 |
| 109 | Q9I4I1\|Q9I4I1_PSEAE | Nucleotide biosynthesis and metabolism | Ribonucleoside-diphosphate reductase | -1.32 | 0.002 |
| 392 | Q9I0M2\|Q9I0M2_PSEAE | Nucleotide biosynthesis and metabolism | Thioredoxin reductase | 1.87 | 0.001 |
| 605 | Q9LBK2\|Q9LBK2_PSEAE | Putative enzymes | (R)-specific enoyl-CoA hydratase | 1.20 | 0.034 |
| 159 | Q9HY63\|ARNA_PSEAE | Putative enzymes | Bifunctional polymyxin resistance protein arnA | 1.28 | 0.001 |
| 319 | Q9I028\|Q9I028_PSEAE | Putative enzymes | Probable acyl-CoA dehydrogenase | 2.08 | 0.026 |
| 99 | P72132\|P72132_PSEAE | Putative enzymes | Probable UDP-glucose/GDP-mannose dehydrogenase WbpA | 1.29 | 0.000 |
| 162 | Q9X2T1\|THIO_PSEAE | Putative enzymes | Thioredoxin | -1.70 | 0.005 |
| 74 | O52760\|RPOA_PSEAE | Transcription, RNA processing and degradation | DNA-directed RNA polymerase subunit alpha | 1.22 | 0.004 |
| 157 | Q9HV46\|GREA_PSEAE | Transcription, RNA processing and degradation | Transcription elongation factor greA | 1.29 | 0.013 |
| 27 | P15276\|ALGP_PSEAE | Transcriptional regulators | Transcriptional regulatory protein algP | 1.10 | 0.030 |
| 7 | Q9HZ71\|RS1_PSEAE | Translation, post-translational modification, degradation | 30S ribosomal protein S1 | 1.19 | 0.009 |
| 70 | Q9HWD4\|RS10_PSEAE | Translation, post-translational modification, degradation | 30S ribosomal protein S10 | -1.63 | 0.000 |
| 84 | Q9HWF8\|RS11_PSEAE | Translation, post-translational modification, degradation | 30S ribosomal protein S11 | -1.33 | 0.033 |
| 79 | Q9HWD0\|RS12_PSEAE | Translation, post-translational modification, degradation | 30S ribosomal protein S12 | 1.41 | 0.011 |
| 373 | Q9HV58\|RS15_PSEAE | Translation, post-translational modification, degradation | 30S ribosomal protein S15 | -1.30 | 0.001 |
| 418 | Q9HWD9\|RS19_PSEAE | Translation, post-translational modification, degradation | 30S ribosomal protein S19 | -2.21 | 0.041 |
| 37 | O82850\|RS2_PSEAE | Translation, post-translational modification, degradation | 30S ribosomal protein S2 | -1.44 | 0.001 |
| 119 | Q9I5V8\|RS21_PSEAE | Translation, post-translational modification, degradation | 30S ribosomal protein S21 | -1.27 | 0.000 |
| 46 | Q9HWE1\|RS3_PSEAE | Translation, post-translational modification, degradation | 30S ribosomal protein S3 | -1.19 | 0.006 |
| 14 | O52759\|RS4_PSEAE | Translation, post-translational modification, degradation | 30S ribosomal protein S4 | -1.20 | 0.009 |
| 95 | Q9HUM9\|RS6_PSEAE | Translation, post-translational modification, degradation | 30S ribosomal protein S6 | -1.81 | 0.003 |
| 52 | Q9HWE9\|RS8_PSEAE | Translation, post-translational modification, degradation | 30S ribosomal protein S8 | -1.84 | 0.000 |
| 117 | Q9HVY3\|RS9_PSEAE | Translation, post-translational modification, degradation | 30S ribosomal protein S9 | -1.51 | 0.000 |
| 57 | Q9HWC5\|RL11_PSEAE | Translation, post-translational modification, degradation | 50S ribosomal protein L11 | -1.40 | 0.000 |
| 171 | Q9HWE2\|RL16_PSEAE | Translation, post-translational modification, degradation | 50S ribosomal protein L16 | -1.52 | 0.001 |
| 250 | Q9HWF1\|RL18_PSEAE | Translation, post-translational modification, degradation | 50S ribosomal protein L18 | -1.41 | 0.001 |
| 69 | Q9HVL6\|RL21_PSEAE | Translation, post-translational modification, degradation | 50S ribosomal protein L21 | -2.09 | 0.000 |
| 229 | Q9HVL7\|RL27_PSEAE | Translation, post-translational modification, degradation | 50S ribosomal protein L27 | -1.37 | 0.012 |
| 164 | Q9HWF3\|RL30_PSEAE | Translation, post-translational modification, degradation | 50S ribosomal protein L30 | -1.48 | 0.003 |
| 96 | Q9HZN4\|RL32_PSEAE | Translation, post-translational modification, degradation | 50S ribosomal protein L32 | 1.42 | 0.000 |
| 359 | P29436\|RL34_PSEAE | Translation, post-translational modification, degradation | 50S ribosomal protein L34 | -1.26 | 0.044 |
| 271 | Q9I0A1\|RL35_PSEAE | Translation, post-translational modification, degradation | 50S ribosomal protein L35 | -1.15 | 0.007 |
| 44 | Q9HWD6\|RL4_PSEAE | Translation, post-translational modification, degradation | 50S ribosomal protein L4 | -1.33 | 0.020 |
| 155 | Q9HWE7\|RL5_PSEAE | Translation, post-translational modification, degradation | 50S ribosomal protein L5 | 14.42 | 0.000 |
| 101 | Q9HWF0\|RL6_PSEAE | Translation, post-translational modification, degradation | 50S ribosomal protein L6 | -1.46 | 0.000 |
| 34 | Q9HWC8\|RL7_PSEAE | Translation, post-translational modification, degradation | 50S ribosomal protein L7/L12 | 1.70 | 0.000 |
| 513 | Q9HVN5\|CLPB_PSEAE | Translation, post-translational modification, degradation | Chaperone protein clpB | 1.33 | 0.003 |
| 390 | Q9HZZ2\|EFP_PSEAE | Translation, post-translational modification, degradation | Elongation factor P | -2.35 | 0.000 |
| 148 | O82851\|EFTS_PSEAE | Translation, post-translational modification, degradation | Elongation factor Ts | -1.24 | 0.005 |
| 127 | Q9HI36\|MAJE_PSEAE | Translation, post-translational modification, degradation | Major exported protein | 1.47 | 0.006 |
| 465 | Q59641\|PPIA_PSEAE | Translation, post-translational modification, degradation | Peptidyl-prolyl cis-trans isomerase A | -1.25 | 0.035 |
| 332 | Q9HWK5\|Q9HWK5_PSEAE | Translation, post-translational modification, degradation | Peptidyl-prolyl cis-trans isomerase C2 | -1.39 | 0.020 |
| 280 | Q9HU50\|Q9HU50_PSEAE | Translation, post-translational modification, degradation | Probable carboxyl-terminal protease | 1.23 | 0.040 |
| 228 | Q9HUM2\|Q9HUM2_PSEAE | Translation, post-translational modification, degradation | Protease subunit HflK | 1.19 | 0.047 |
| 215 | P0C2B2\|DSBA_PSEAE | Translation, post-translational modification, degradation | Thiol:disulfide interchange protein dsbA | -2.39 | 0.000 |
| 133 | O50181\|O50181_PSEAE | Transport of small molecule | Arginine and ornithine binding protein | -1.31 | 0.007 |
| 5 | P13794\|PORF_PSEAE | Transport of small molecule | Outer membrane porin F | -1.13 | 0.050 |
| 121 | Q9HZ51\|Q9HZ51_PSEAE | Transport of small molecule | Probable ATP-binding component of ABC transpor | -1.41 | 0.003 |
| 321 | Q9I1I4\|Q9I1I4_PSEAE | Transport of small molecule | Probable glucose-sensitive porin | -2.49 | 0.012 |
| 329 | Q9I6K7\|Q9I6K7_PSEAE | Transport of small molecule | Sulfate-binding protein | -1.39 | 0.044 |
| 126 | Q9I0H9\|Q9I0H9_PSEAE | Unknown | Putative uncharacterized protein | -2.57 | 0.000 |
| 264 | Q9HXU9\|Q9HXU9_PSEAE | Unknown | Putative uncharacterized protein | -1.97 | 0.005 |
| 336 | Q9I4S1\|Q9I4S1_PSEAE | Unknown | Putative uncharacterized protein | -1.46 | 0.005 |
| 112 | Q9HV60\|Q9HV60_PSEAE | Unknown | Putative uncharacterized protein | -1.36 | 0.008 |
| 458 | Q9I368\|Q9I368_PSEAE | Unknown | Putative uncharacterized protein | -1.33 | 0.042 |
| 600 | Q9HVZ2\|Q9HVZ2_PSEAE | Unknown | Putative uncharacterized protein | -1.30 | 0.023 |
| 582 | Q9I574\|Q9I574_PSEAE | Unknown | Putative uncharacterized protein | -1.29 | 0.012 |
| 620 | Q9HWP2\|Q9HWP2_PSEAE | Unknown | Putative uncharacterized protein | -1.27 | 0.005 |
| 10 | Q9I5F7\|Q9I5F7_PSEAE | Unknown | Putative uncharacterized protein | -1.19 | 0.029 |
| 592 | Q9HVI2\|Q9HVI2_PSEAE | Unknown | Putative uncharacterized protein | 1.21 | 0.002 |
| 351 | O68801\|O68801_PSEAE | Unknown | Putative uncharacterized protein | 1.27 | 0.000 |
| 609 | Q9HZ38\|Q9HZ38_PSEAE | Unknown | Putative uncharacterized protein | 1.35 | 0.007 |
| 11 | Q9I762\|Q9I762_PSEAE | Unknown | Putative uncharacterized protein | 1.35 | 0.000 |
| 210 | Q9HW49\|Q9HW49_PSEAE | Unknown | Putative uncharacterized protein | 1.36 | 0.040 |
| 489 | Q9I755\|Q9I755_PSEAE | Unknown | Putative uncharacterized protein | 1.43 | 0.005 |
| 531 | Q9I520\|Q9I520_PSEAE | Unknown | Putative uncharacterized protein | 1.44 | 0.022 |
| 53 | Q9I367\|Q9I367_PSEAE | Unknown | Putative uncharacterized protein | 1.46 | 0.032 |
| 401 | Q9HYT1\|Q9HYT1_PSEAE | Unknown | Putative uncharacterized protein | 1.49 | 0.020 |
| 227 | Q9HVX4\|Q9HVX4_PSEAE | Unknown | Putative uncharacterized protein | 1.52 | 0.002 |
| 188 | Q9I5U9\|Q9I5U9_PSEAE | Unknown | Putative uncharacterized protein | 1.58 | 0.030 |
| 613 | Q9HVA9\|Q9HVA9_PSEAE | Unknown | Putative uncharacterized protein | 2.58 | 0.022 |
| 400 | Q9I3A4\|Y1618_PSEAE | Unknown | UPF0152 protein PA1618 | -2.47 | 0.000 |
| 32 | Q9HVT2\|Y4489_PSEAE | Unknown | UPF0192 protein PA4489 | 1.23 | 0.048 |
