# Supplementary material for: Glucose Starvation-Induced Dispersal of Pseudomonas aeruginosa Biofilms Is cAMP and Energy Dependent
Source: PLoS One. 2012 Aug 14;7(8):e42874. doi: 10.1371/journal.pone.0042874 (PMC3419228; doi:10.1371/journal.pone.0042874)
Supplement: Table S2 — Differentially expression proteins (109) with p -value<0.05 showing relative fold change from the starved effluent samples compared to the non-starved effluent samples. (DOCX) [file pone.0042874.s002.docx]

**Table S2. Differentially expression proteins (109) with *p*-value < 0.05 showing relative fold change from the starved effluent samples compared to the non-starved effluent samples.**

| *N* | *Accession* | *Function* | *Name* | *Fold-Change* | *P-value* |
| --- | --- | --- | --- | --- | --- |
| 195 | Q9HW91\|Q9HW91_PSEAE | Adaptation, Protection | Chemotactic transducer PctB | 1.15 | 0.022 |
| 87 | Q9I2T9\|Q9I2T9_PSEAE | Adaptation, Protection | Lon protease | -1.25 | 0.038 |
| 63 | Q51548\|PVDA_PSEAE | Adaptation, Protection | L-ornithine 5-monooxygenase | -1.46 | 0.000 |
| 185 | Q9I4H8\|Q9I4H8_PSEAE | Adaptation, Protection | Probable cold-shock protein | -1.54 | 0.000 |
| 350 | P57668\|TPX_PSEAE | Adaptation, Protection | Probable thiol peroxidase | 1.21 | 0.024 |
| 16 | Q9I157\|Q9I157_PSEAE | Adaptation, Protection | Pyoverdine synthetase PvdL | -1.33 | 0.003 |
| 238 | Q9I182\|Q9I182_PSEAE | Adaptation, Protection | Pyoverdine synthetase D | -1.23 | 0.026 |
| 389 | Q9I184\|Q9I184_PSEAE | Adaptation, Protection | Pyoverdine synthetase F | 1.60 | 0.013 |
| 72 | Q9I3D3\|Q9I3D3_PSEAE | Amino acid biosynthesis and metabolism | 2-oxoglutarate dehydrogenase | -1.35 | 0.000 |
| 19 | P57703\|METE_PSEAE | Amino acid biosynthesis and metabolism | 5-methyltetrahydropteroyltriglutamate-hh  hohhh homocysteine methyltransferase-homocys ??? | -2.07 | 0.000 |
|  |  |  | homocysteine methyltransferase |  |  |
| 218 | Q9I344\|AROC_PSEAE | Amino acid biosynthesis and metabolism | Chorismate synthase | -1.15 | 0.010 |
| 86 | Q9I6H5\|Q9I6H5_PSEAE | Amino acid biosynthesis and metabolism | D-3-phosphoglycerate dehydrogenase | -1.34 | 0.000 |
| 89 | Q9I3D1\|DLDH2_PSEAE | Amino acid biosynthesis and metabolism | Dihydrolipoamide dehydrogenase | -1.20 | 0.002 |
| 20 | Q9HU65\|GLNA_PSEAE | Amino acid biosynthesis and metabolism | Glutamine synthetase | -1.18 | 0.001 |
| 154 | Q51510\|Q51510_PSEAE | Amino acid biosynthesis and metabolism | Protein E1 | 1.37 | 0.005 |
| 66 | Q9I5Z0\|METK_PSEAE | Amino acid biosynthesis and metabolism | S-adenosylmethionine synthetase | -1.47 | 0.000 |
| 30 | Q9HVI7\|GLYA3_PSEAE | Amino acid biosynthesis and metabolism | Serine hydroxymethyltransferase 3 | -1.29 | 0.000 |
| 347 | O54438\|FABG_PSEAE | Biosynthesis of cofactors, prosthetic groups and carriers | 3-oxoacyl-[acyl-carrier-protein] reductase | 1.23 | 0.005 |
| 140 | Q9HVC5\|KPRS_PSEAE | Carbon compound catabolism | Ribose-phosphate pyrophosphokinase | 1.30 | 0.029 |
| 204 | Q9HVU0\|Q9HVU0_PSEAE | Cell division | Rod shape-determining protein MreB | 1.22 | 0.016 |
| 528 | Q9HT12\|Q9HT12_PSEAE | Cell division | Chromosome partitioning protein Spo0J | -1.55 | 0.029 |
| 122 | Q9I7C4\|DPO3B_PSEAE | DNA replication, recombination, modification and repair | DNA polymerase III subunit beta | 1.24 | 0.038 |
| 28 | P05384\|DBHB_PSEAE | DNA replication, recombination, modification and repair | DNA-binding protein HU-beta | -1.24 | 0.000 |
| 535 | Q9I3F5\|ACON1_PSEAE | Energy metabolism | Aconitate hydratase 1 | 2.12 | 0.001 |
| 474 | Q9HT17\|ATPD_PSEAE | Energy metabolism | ATP synthase delta chain | 1.69 | 0.002 |
| 113 | P00282\|AZUR_PSEAE | Energy metabolism | Azurin | 1.22 | 0.000 |
| 151 | Q51404\|FUMC1_PSEAE | Energy metabolism | Fumarate hydratase class II 1 | -1.32 | 0.031 |
| 94 | P24474\|NIRS_PSEAE | Energy metabolism | Nitrite reductase | 1.39 | 0.002 |
| 71 | Q9HUC3\|Q9HUC3_PSEAE | Energy metabolism | Polyhydroxyalkanoate synthesis protein PhaF | 1.17 | 0.032 |
| 393 | Q9I3G2\|Q9I3G2_PSEAE | Energy metabolism | Probable cytochrome c | 1.84 | 0.001 |
| 291 | Q9HYF4\|MQO1_PSEAE | Energy metabolism | Probable malate:quinone oxidoreductase 1 | -1.34 | 0.010 |
| 42 | Q9HXU8\|Q9HXU8_PSEAE | Energy metabolism | Probable outer membrane protein | 1.13 | 0.010 |
| 323 | Q9I3D4\|Q9I3D4_PSEAE | Energy metabolism | Succinate dehydrogenase | 2.50 | 0.000 |
| 40 | Q51567\|SUCD_PSEAE | Energy metabolism | Succinyl-CoA ligase [ADP-forming] subunit alpha | -1.31 | 0.000 |
| 135 | Q9HZJ2\|FADB_PSEAE | Fatty acid and phospholipid metabolism | Fatty acid oxidation complex subunit alpha | -1.18 | 0.016 |
| 73 | Q9HW32\|Q9HW32_PSEAE | Membrane proteins | Insulin-cleaving metalloproteinase outer membrane | -1.19 | 0.009 |
| 39 | P11221\|OPRI_PSEAE | Membrane proteins | Major outer membrane lipoprotein | 1.42 | 0.000 |
| 5 | P13794\|PORF_PSEAE | Membrane proteins | Outer membrane porin F | 2.14 | 0.000 |
| 51 | Q9HWW1\|Q9HWW1_PSEAE | Membrane proteins | Outer membrane protein OprG | 1.51 | 0.000 |
| 108 | Q9I4Z4\|PAL_PSEAE | Membrane proteins | Peptidoglycan-associated lipoprotein | 2.09 | 0.000 |
| 326 | Q9HZU7\|Q9HZU7_PSEAE | Membrane proteins | Probable outer membrane protein | -1.23 | 0.009 |
| 9 | Q9HUX3\|Q9HUX3_PSEAE | Membrane proteins | Probable outer membrane protein | 1.51 | 0.000 |
| 114 | Q9I083\|Q9I083_PSEAE | Membrane proteins | Probable outer membrane protein | 1.75 | 0.000 |
| 298 | Q9I696\|Q9I696_PSEAE | Motility & Attachment | component of chemotactic signal transduction system | 1.24 | 0.019 |
| 35 | P34750\|PILQ_PSEAE | Motility & Attachment | Fimbrial assembly protein pilQ | 1.90 | 0.000 |
| 177 | Q51351\|Q51351_PSEAE | Motility & Attachment | PilM protein | 1.77 | 0.042 |
| 295 | Q9HXZ4\|PYRG_PSEAE | Nucleotide biosynthesis and metabolism | CTP synthase | -1.29 | 0.001 |
| 138 | Q9HXM5\|Q9HXM5_PSEAE | Nucleotide biosynthesis and metabolism | Inosine-5'-monophosphate dehydrogenase | -1.19 | 0.002 |
| 173 | Q9HXN2\|PUR4_PSEAE | Nucleotide biosynthesis and metabolism | Phosphoribosylformylglycinamidine synthase | -1.50 | 0.003 |
| 159 | Q9HY63\|ARNA_PSEAE | Putative enzymes | Bifunctional polymyxin resistance protein arnA | -1.99 | 0.000 |
| 343 | Q9I0K4\|ACEA_PSEAE | Putative enzymes | Isocitrate lyase | 1.13 | 0.048 |
| 278 | Q9I612\|Q9I612_PSEAE | Putative enzymes | Probable acyl-CoA dehydrogenase | -1.25 | 0.013 |
| 162 | Q9X2T1\|THIO_PSEAE | Putative enzymes | Thioredoxin | 1.36 | 0.016 |
| 99 | P72132\|P72132_PSEAE | Putative enzymes | WbpA | -1.16 | 0.021 |
| 432 | O85739\|O85739_PSEAE | Secreted Factors (toxins, enzymes, alginate) | dihydroaeruginoic acid synthetase | -1.61 | 0.032 |
| 263 | O68822\|AMPA_PSEAE | Transcription, RNA processing and degradation | Cytosol aminopeptidase | 1.53 | 0.001 |
| 74 | O52760\|RPOA_PSEAE | Transcription, RNA processing and degradation | DNA-directed RNA polymerase subunit alpha | -1.11 | 0.009 |
| 3 | Q51561\|RPOB_PSEAE | Transcription, RNA processing and degradation | DNA-directed RNA polymerase subunit beta | -1.12 | 0.004 |
| 429 | P38108\|MUCB_PSEAE | Transcriptional regulators | Sigma factor algU regulatory protein mucB | 1.62 | 0.043 |
| 27 | P15276\|ALGP_PSEAE | Transcriptional regulators | Transcriptional regulatory protein algP | -1.23 | 0.000 |
| 70 | Q9HWD4\|RS10_PSEAE | Translation, post-translational modification, degradation | 30S ribosomal protein S10 | 1.24 | 0.000 |
| 84 | Q9HWF8\|RS11_PSEAE | Translation, post-translational modification, degradation | 30S ribosomal protein S11 | 1.21 | 0.026 |
| 79 | Q9HWD0\|RS12_PSEAE | Translation, post-translational modification, degradation | 30S ribosomal protein S12 | -1.38 | 0.000 |
| 107 | Q9HWE8\|RS14_PSEAE | Translation, post-translational modification, degradation | 30S ribosomal protein S14 | 1.43 | 0.044 |
| 469 | Q9HXP9\|RS16_PSEAE | Translation, post-translational modification, degradation | 30S ribosomal protein S16 | 1.43 | 0.001 |
| 100 | Q9HWE4\|RS17_PSEAE | Translation, post-translational modification, degradation | 30S ribosomal protein S17 | -1.13 | 0.008 |
| 149 | Q9HUN0\|RS18_PSEAE | Translation, post-translational modification, degradation | 30S ribosomal protein S18 | 1.20 | 0.026 |
| 418 | Q9HWD9\|RS19_PSEAE | Translation, post-translational modification, degradation | 30S ribosomal protein S19 | 1.54 | 0.016 |
| 37 | O82850\|RS2_PSEAE | Translation, post-translational modification, degradation | 30S ribosomal protein S2 | 1.39 | 0.000 |
| 56 | Q9HWD1\|RS7_PSEAE | Translation, post-translational modification, degradation | 30S ribosomal protein S7 | 1.11 | 0.013 |
| 117 | Q9HVY3\|RS9_PSEAE | Translation, post-translational modification, degradation | 30S ribosomal protein S9 | 1.45 | 0.000 |
| 171 | Q9HWE2\|RL16_PSEAE | Translation, post-translational modification, degradation | 50S ribosomal protein L16 | 1.40 | 0.000 |
| 31 | Q9HWD8\|RL2_PSEAE | Translation, post-translational modification, degradation | 50S ribosomal protein L2 | 1.11 | 0.036 |
| 69 | Q9HVL6\|RL21_PSEAE | Translation, post-translational modification, degradation | 50S ribosomal protein L21 | 1.22 | 0.000 |
| 29 | Q9HWD5\|RL3_PSEAE | Translation, post-translational modification, degradation | 50S ribosomal protein L3 | -1.15 | 0.002 |
| 131 | Q9HY25\|RL31B_PSEAE | Translation, post-translational modification, degradation | 50S ribosomal protein L31 type B | -1.19 | 0.002 |
| 96 | Q9HZN4\|RL32_PSEAE | Translation, post-translational modification, degradation | 50S ribosomal protein L32 | -1.47 | 0.000 |
| 271 | Q9I0A1\|RL35_PSEAE | Translation, post-translational modification, degradation | 50S ribosomal protein L35 | 1.20 | 0.046 |
| 34 | Q9HWC8\|RL7_PSEAE | Translation, post-translational modification, degradation | 50S ribosomal protein L7/L12 | -1.46 | 0.000 |
| 361 | O69078\|CSRA_PSEAE | Translation, post-translational modification, degradation | Carbon storage regulator homolog | 1.26 | 0.006 |
| 1 | Q9HWD2\|EFG1_PSEAE | Translation, post-translational modification, degradation | Elongation factor G 1 | 1.22 | 0.000 |
| 390 | Q9HZZ2\|EFP_PSEAE | Translation, post-translational modification, degradation | Elongation factor P | 1.80 | 0.000 |
| 105 | Q9HXZ5\|ENO_PSEAE | Translation, post-translational modification, degradation | Enolase | 1.31 | 0.003 |
| 280 | Q9HU50\|Q9HU50_PSEAE | Translation, post-translational modification, degradation | Probable carboxyl-terminal protease | -1.14 | 0.024 |
| 215 | P0C2B2\|DSBA_PSEAE | Translation, post-translational modification, degradation | Thiol:disulfide interchange protein dsbA | 1.41 | 0.010 |
| 346 | P65116\|IF1_PSEAE | Translation, post-translational modification, degradation | Translation initiation factor IF-1 | 1.28 | 0.035 |
| 256 | Q9HWG3\|Q9HWG3_PSEAE | Transport of small molecules | Pyochelin biosynthesis protein PchD | -1.76 | 0.049 |
| 269 | Q9I6J1\|Q9I6J1_PSEAE | Transport of small molecules | Polyamine transport protein | 1.27 | 0.036 |
| 570 | Q9HT81\|ENGB_PSEAE | Unknown | Conserved hypothetical protein | 1.18 | 0.039 |
| 531 | Q9I520\|Q9I520_PSEAE | Unknown | Putative uncharacterized protein | -1.77 | 0.010 |
| 304 | Q9HZI5\|Q9HZI5_PSEAE | Unknown | Putative uncharacterized protein | -1.48 | 0.026 |
| 199 | Q9HT34\|Q9HT34_PSEAE | Unknown | Putative uncharacterized protein | -1.48 | 0.005 |
| 552 | Q9HXG8\|Q9HXG8_PSEAE | Unknown | Putative uncharacterized protein | -1.43 | 0.048 |
| 112 | Q9HV60\|Q9HV60_PSEAE | Unknown | Putative uncharacterized protein | -1.29 | 0.000 |
| 423 | Q9HT39\|Q9HT39_PSEAE | Unknown | Putative uncharacterized protein | -1.13 | 0.013 |
| 354 | Q9HZU6\|Q9HZU6_PSEAE | Unknown | Putative uncharacterized protein | -1.07 | 0.018 |
| 600 | Q9HVZ2\|Q9HVZ2_PSEAE | Unknown | Putative uncharacterized protein | 1.11 | 0.049 |
| 11 | Q9I762\|Q9I762_PSEAE | Unknown | Putative uncharacterized protein | 1.12 | 0.048 |
| 10 | Q9I5F7\|Q9I5F7_PSEAE | Unknown | Putative uncharacterized protein | 1.20 | 0.000 |
| 542 | Q9I749\|Q9I749_PSEAE | Unknown | Putative uncharacterized protein | 1.22 | 0.001 |
| 25 | Q9HYT5\|Q9HYT5_PSEAE | Unknown | Putative uncharacterized protein | 1.23 | 0.030 |
| 592 | Q9HVI2\|Q9HVI2_PSEAE | Unknown | Putative uncharacterized protein | 1.24 | 0.017 |
| 53 | Q9I367\|Q9I367_PSEAE | Unknown | Putative uncharacterized protein | 1.28 | 0.002 |
| 561 | Q9I129\|Q9I129_PSEAE | Unknown | Putative uncharacterized protein | 1.37 | 0.009 |
| 126 | Q9I0H9\|Q9I0H9_PSEAE | Unknown | Putative uncharacterized protein | 1.38 | 0.001 |
| 458 | Q9I368\|Q9I368_PSEAE | Unknown | Putative uncharacterized protein | 1.53 | 0.004 |
| 351 | O68801\|O68801_PSEAE | Unknown | Putative uncharacterized protein | 1.55 | 0.000 |
| 400 | Q9I3A4\|Y1618_PSEAE | Unknown | UPF0152 protein PA1618 | 1.47 | 0.005 |
| 453 | P33641\|Y9F5_PSEAE | Unknown | UPF0169 lipoprotein PA4545 | -1.26 | 0.020 |
