# Supplementary material for: Glucose Starvation-Induced Dispersal of Pseudomonas aeruginosa Biofilms Is cAMP and Energy Dependent
Source: PLoS One. 2012 Aug 14;7(8):e42874. doi: 10.1371/journal.pone.0042874 (PMC3419228; doi:10.1371/journal.pone.0042874)
Supplement: Table S3 — Overlap of proteins identified in Tables S1 and S2 for biofilm and planktonic cells. (DOCX) [file pone.0042874.s003.docx]

**Table S3. Overlap of proteins identified in Tables S1 and S2 for biofilm and planktonic cells.**

| *Accession* | *Overlap proteins* | *Function* | *Biofilm population/  Fold change* | *P-value* | *Planktonic population/  Fold change* | *P-value* |
| --- | --- | --- | --- | --- | --- | --- |
| Q51548\|PVDA_PSEAE | L-ornithine 5-monooxygenase | Adaptation, Protection | 1.35 | 0.003 | -1.46 | 0 |
| Q9I4H8\|Q9I4H8_PSEAE | Probable cold-shock protein | Adaptation, Protection | 1.26 | 0.044 | -1.54 | 0 |
| P57668\|TPX_PSEAE | Probable thiol peroxidase | Adaptation, Protection | -1.37 | 0.034 | 1.21 | 0.024 |
| Q9I157\|Q9I157_PSEAE | Pyoverdine synthase PvdL | Adaptation, Protection | 1.16 | 0.034 | -1.33 | 0.003 |
| Q9I3D4\|Q9I3D4_PSEAE | Succinate dehydrogenase | Energy metabolism | 3.7 | 0 | 2.5 | 0 |
| Q9I4Z4\|PAL_PSEAE | Peptidoglycan-associated lipoprotein | Membrane proteins | 4.61 | 0 | 2.09 | 0 |
| Q9HY63\|ARNA_PSEAE | Bifunctional polymyxin resistance protein arnA | Putative enzymes | 1.28 | 0.001 | -1.99 | 0 |
| Q9I028\|Q9I028_PSEAE | Probable acyl-CoA dehydrogenase | Putative enzymes | 2.08 | 0.026 | -1.25 | 0.013 |
| Q9X2T1\|THIO_PSEAE | Thioredoxin | Putative enzymes | -1.7 | 0.005 | 1.36 | 0.016 |
| O52760\|RPOA_PSEAE | DNA-directed RNA polymerase subunit alpha | Transcription, RNA processing and degradation | 1.22 | 0.004 | -1.12 | 0.009 |
| Q9HWD4\|RS10_PSEAE | Transcriptional regulatory protein algP | Transcriptional regulators | 1.1 | 0 | -1.23 | 0 |
| Q9HWF8\|RS11_PSEAE | 30S ribosomal protein S10 | Translation, post-translational modification, degradation | -1.63 | 0.033 | 1.24 | 0 |
| Q9HWD0\|RS12_PSEAE | 30S ribosomal protein S11 | Translation, post-translational modification, degradation | -1.33 | 0.011 | 1.21 | 0.026 |
| Q9HWD0\|RS12_PSEAE | 30S ribosomal protein S12 | Translation, post-translational modification, degradation | 1.41 | 0.011 | -1.38 | 0 |
| Q9HWD9\|RS19_PSEAE | 30S ribosomal protein S19 | Translation, post-translational modification, degradation | -2.21 | 0.041 | 1.54 | 0.016 |
| O82850\|RS2_PSEAE | 30S ribosomal protein S2 | Translation, post-translational modification, degradation | -1.44 | 0.001 | 1.39 | 0 |
| Q9HVY3\|RS9_PSEAE | 30S ribosomal protein S9 | Translation, post-translational modification, degradation | -1.51 | 0 | 1.45 | 0 |
| Q9HWE2\|RL16_PSEAE | 50S ribosomal protein L16 | Translation, post-translational modification, degradation | -1.52 | 0.001 | 1.4 | 0 |
| Q9HVL6\|RL21_PSEAE | 50S ribosomal protein L21 | Translation, post-translational modification, degradation | -2.09 | 0 | 1.22 | 0 |
| Q9HWC8\|RL7_PSEAE | 50S ribosomal protein L7/L12 | Translation, post-translational modification, degradation | 1.7 | 0 | -1.46 | 0 |
| Q9HZZ2\|EFP_PSEAE | Elongation factor P | Translation, post-translational modification, degradation | -2.35 | 0 | 1.8 | 0 |
| Q9HU50\|Q9HU50_PSEAE | Probable carboxyl-terminal protease | Translation, post-translational modification, degradation | 1.23 | 0.04 | -1.14 | 0.024 |
| P0C2B2\|DSBA_PSEAE | Thiol:disulfide interchange protein dsbA | Translation, post-translational modification, degradation | -2.39 | 0 | 1.41 | 0.01 |
